# An accelerated Rauhut–Currier dimerization enabled the synthesis of (±)-incarvilleatone and anticancer studies

**DOI:** 10.3762/bjoc.19.19

**Published:** 2023-02-21

**Authors:** Tharun K Kotammagari, Sweta Misra, Sayantan Paul, Sunita Kunte, Rajesh G Gonnade, Manas K Santra, Asish K Bhattacharya

**Affiliations:** 1 Division of Organic Chemistry, CSIR-National Chemical Laboratory, Dr. Homi Bhabha Road, Pune-411 008, India; 2 Academy of Scientific and Innovative Research (AcSIR), CSIR-HRDC Campus, Sector 19, Kamla Nehru Nagar, Ghaziabad, UP, 201 002, India; 3 Cancer Biology Division, National Centre for Cell Sciences, Ganesh Khind Road, Pune-411 007, India; 4 Centre for Material Characterization, CSIR-National Chemical Laboratory, Dr. Homi Bhabha Road, Pune-411 008, India

**Keywords:** dimerization, incarviditone, incarvilleatone, oxa-Michael, Rauhut–Currier

## Abstract

The total synthesis of racemic incarvilleatone has been achieved by utilizing unexplored accelerated Rauhut–Currier (RC) dimerization. The other key steps of the synthesis are oxa-Michael and aldol reactions in a tandem sequence. Racemic incarvilleatone was separated by chiral HPLC and the configuration of each enantiomer was determined by single-crystal X-ray analysis. In addition, a one-pot synthesis of (±)-incarviditone has been achieved from *rac*-rengyolone by using KHMDS as a base. We have also assessed the anticancer activity of all the synthesized compounds in breast cancer cells nonetheless, they exhibited very limited growth suppression activity.

## Introduction

**Figure 1:**
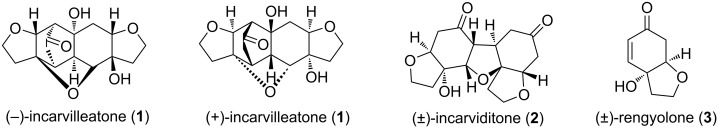
Structures of (±)-incarvilleatone (**1**), (±)-incarviditone (**2**), and (±)-rengyolone (**3**).

(±)-Incarvilleatone (**1**) is a dimeric cyclohexylethanoid isolated by Zhang and co-workers [1] in racemic form from the Chinese plant *Incarvillea younghusbandii* (Figure 1). This plant is used in Chinese folk medicine to treat dizziness and anemia [1].

Zhang and co-workers [1] separated the racemic incarvilleatone in two individual enantiomers, (−)-incarvilleatone [(−)-**1**] and (+)-incarvilleatone [(+)-**1**] by performing chiral HPLC. The structure of *rac*-incarvilleatone (**1**) was determined by spectroscopic methods and single crystal X-ray analysis. However, they were unable to obtain single crystals of either of the enantiomers (−)-incarvilleatone [(−)-**1**] and (+)-incarvilleatone [(+)-**1**]. (±)-Incarviditone (**2**), a novel benzofuranone dimer was isolated from *I. delavayi* along with known (±)-rengyolone (**3**) [2]. (±)-Incarviditone (**2**) presents a new carbon-skeleton by being the first benzofuranone dimer connected by a C–C bond. The cytotoxicity of (±)-incarviditone (**2**) has been assayed against cell lines A549, LOVO, HL-60, 6TCEM, and HepG2 and displayed cytotoxicity against the HL-60 cell line with an IC_50_ value of 14.8 mg/mL and against the 6T-CEM cell line with an IC_50_ value of 22.2 mg/mL.

Lawrence et al. [3] and Tang et al. [4] have reported the synthesis of (±)-incarvilleatone (**1**) and (±)-incarviditone (**2**) via biomimetic dimerization of (±)-rengyolone (**3**). Despite of these syntheses, the synthesis of the dimeric natural product (±)-incarvilleatone (**1**), whose monomeric unit (±)-rengyolone (**3**) was connected by accelerated intermolecular Rauhut–Currier (RC) reaction was not reported in literature. Herein, we present the total synthesis of (±)-incarvilleatone (**1**) by utilizing an unexplored accelerated Rauhut-Currier (RC) dimerization as a key step. In addition to that HPLC separation of (±)-incarvilleatone (**1**) into its enantiomers (−)-incarvilleatone (−)-**1** and (+)-incarvilleatone (+)-**1** and their X-ray crystallographic analysis which has not yet been reported in the literature is described. The reaction was discovered by Rauhut and Currier [5] in the year 1963. It is a nucleophile-catalyzed C–C bond-forming reaction between two Michael acceptors. This reaction provides access to diverse classes of densely functionalized molecules. Rauhut–Currier (RC) dimerization has some limitations, such as its controlling selectivity for intermolecular reactions in differently activated alkenes, and low reactivity. Han and co-workers [6] addressed the latter one by designing a substrate in which nucleophile functionality is also present in the Michael acceptor to accelerate the reaction. In conventional intermolecular RC reactions, the reaction proceeds by the intermolecular addition of a nucleophilic catalyst to the enone substrate to generate an enolate in the first step. In the second step the enolate ion attacks the other Michael acceptor at β-position in an intermolecular fashion to form a C–C bond between the two Michael acceptors.

**Scheme 1:**
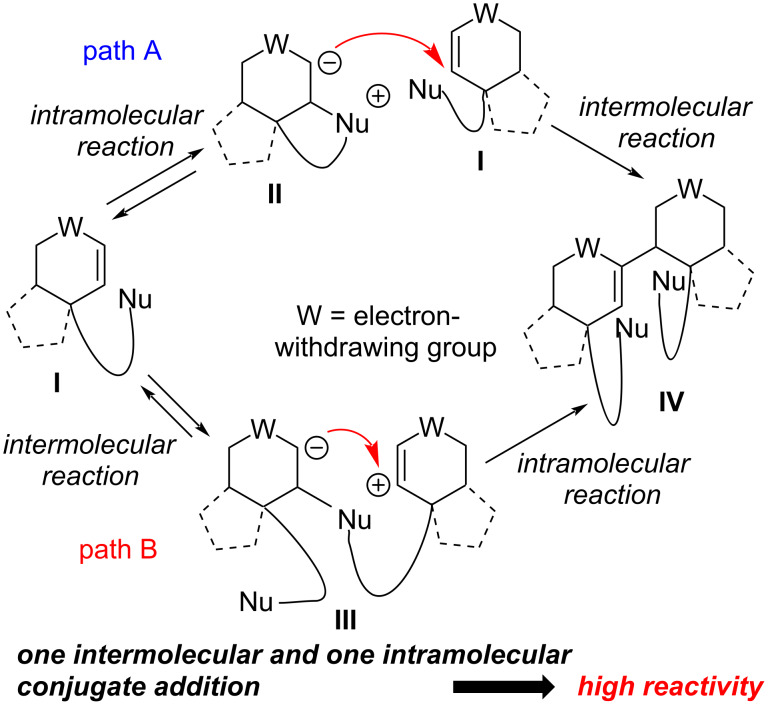
Possible modes of accelerated intermolecular RC reaction, drawn according to [6].

This whole process involves two intermolecular conjugate additions, which leads to low reactivity. In case of intramolecular RC reactions, a high reactivity is observed. This is due to one intermolecular and one intramolecular conjugate addition reaction involved. The low reactivity of intermolecular RC reactions can be improved by incorporating the nucleophilic functionality within a molecule like **I** (Scheme 1).

This nucleophilic functionality present within the enone system first undergoes an intramolecular conjugate addition and is followed by an intermolecular conjugate addition to form a C–C bond (path A). In an alternative approach (path B) first **I** undergoes a nucleophilic conjugate addition in intermolecular fashion to give intermediate **III** and followed by an intramolecular addition to give compound **IV**. In both paths, the involvement of one intramolecular and one intermolecular conjugate addition reaction leads to notable high acceleration in RC reactions.

Based on the accelerated intermolecular Rauhut–Currier reaction reported in the literature [7–9] and our interest in the synthesis of dimeric complex natural products [10], we designed a synthetic scheme for the synthesis of (±)-incarvilleatone (**1**) and (±)-incarviditone (**2**) starting from (±)-rengyolone **3**. We anticipated that (±)-rengyolone (**3**) will be a good substrate to test the accelerated intermolecular Rauhut–Currier reaction. The presence of a nucleophilic functionality (hydroxy group) and an enone system within the same molecule are needed to accelerate the intermolecular RC reaction.

## Results and Discussion

**Scheme 2:**
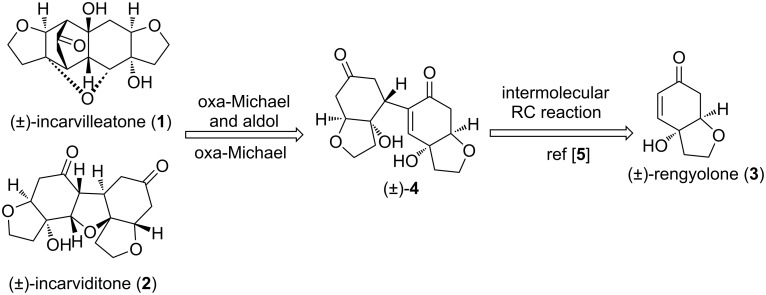
Retrosynthetic plan for the synthesis of (±)-incarvilleatone (**1**) and (±)-incarviditone (**2**).

A retrosynthetic plan for the synthesis of (±)-incarvilleatone (**1**) and (±)-incarviditone (**2**) is delineated in Scheme 2. We envisaged that both the natural products (±)-incarvilleatone (**1**) and (±)-incarviditone (**2**) could be obtained from the RC product **4** by using oxa-Michael and aldol reactions [3–4]. The RC product **4** in turn can be obtained from a monomeric Michael acceptor, i.e., (±)-rengyolone (**3**).

**Scheme 3:**
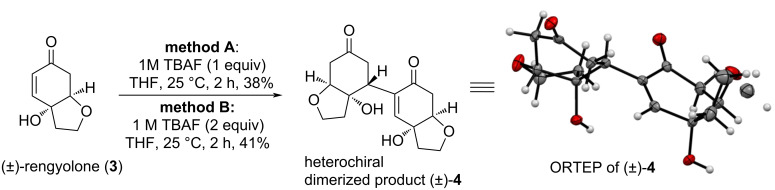
Synthesis of RC dimerized product (±)-**4**.

The Michael acceptor for the intermolecular Rauhut–Currier (RC) reaction, (±)-rengyolone (**3**), was synthesized following a literature procedure [4]. With (±)-rengyolone (**3**), i.e., the monomeric Michael acceptor in hand, we attempted for Rauhut–Currier dimerization. We found out that treatment of (±)-rengyolone (**3**) with 1.0 M TBAF (1 equiv) in THF at room temperature resulted in the formation of heterodimerized product (±)-**4** in 38% yield as a pale-yellow solid (Scheme 3, method A).

**Scheme 4:**
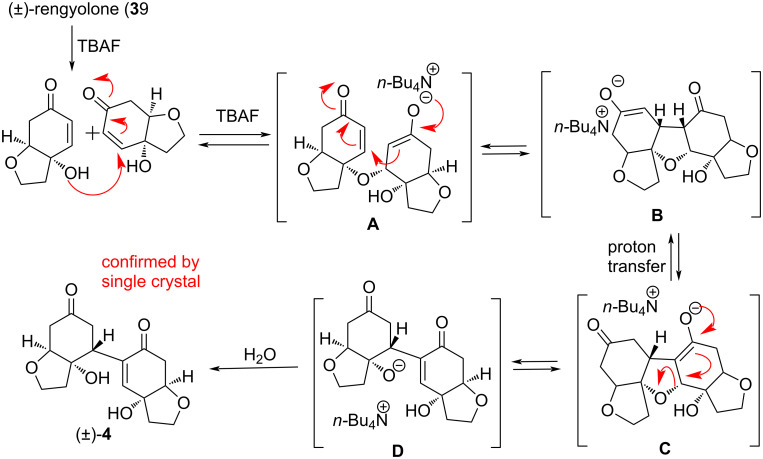
Proposed reaction mechanism for the formation of compound (±)-**4** under TBAF-mediated Rauhut–Currier reaction.

The yield of this reaction could not be improved even by prolonging the reaction time (up to 24 h). However, when we treated (±)-rengyolone (**3**) with 1.0 M TBAF (2 equiv) in THF at room temperature, we obtained the dimeric product (±)-**4** in 41% yield. The formation of dihydroxy dimerized RC product (±)-**4** was confirmed by NMR spectra. In the ^1^H NMR spectrum, the olefin proton was observed at δ 6.75 ppm as a singlet, and the two hydroxy protons were observed at δ 5.60 (s, 1H) and 5.03 (s, 1H) ppm, respectively. In the ^13^C NMR, the two carbonyl groups appeared at δ 197.4 and δ 209.4 ppm, and the corresponding two olefinic carbons were observed at δ 135.7 and δ 148.5 ppm. The formation of the dihydroxy compound (±)-**4** was also confirmed with a D_2_O shake experiment. When we added a drop of D_2_O to the ^1^H NMR sample, the peaks corresponding to the two hydroxy groups were completely absent at δ 5.60 (s, 1H) and 5.03 (s, 1H) ppm (Figure S1, Supporting Information File 1). The formation of the dihydroxy dimeric compound (±)-**4** was further confirmed by HRMS, which showed a peak at 331.1150 corresponding to the C_16_H_20_O_6_Na [M + Na]^+^ ion. After some efforts, to our delight, we could obtain single crystals of (±)-**4** using EtOAc as a solvent. Finally, the formation of heterodimerized dihydroxy RC product (±)-**4** was confirmed by single-crystal X-ray analysis [16]. It is pertinent to mention here that in this reaction we obtained the heterochiral dimerized product (±)-**4**. We did not observe any homochiral dimerized product formation under TBAF reaction conditions. A plausible mechanism for the formation of heterochiral dimerized dihydroxy RC product (±)-**4** through accelerated RC [6] is outlined in Scheme 4.

We proposed that (±)-rengyolone (**3**) undergoes a hydroxy-directed intermolecular conjugate addition [11–15] with another equivalent of (±)-rengyolone (**3**) (enone system) to highly selectively afford intermediate **A**. Via a rapid intramolecular Michael addition of enolate intermediate **A** tetrahydrofuran intermediate **B** with *cis*-fused ring systems is formed as seen in the existing literature [7]. A proton transfer of enolate moiety **B** yields another enolate **C** followed by the β-alkoxy elimination [17] of intermediate **C** to form intermediate **D**. The intermediate **D** on protonation leads to the dihydroxy RC product (±)-**4**. Synthesis of dihydroxy product (±)-**4** was carried out at gram-scale utilizing the key accelerated RC dimerization. In order to synthesize (±)-incarvilleatone (**1**) first, we needed to perform an oxa-Michael addition reaction. For this, we screened various bases [11–15] such as NaHMDS, DBU, NaH, DABCO, *t*-BuOK, aq NaOH but none of them gave the desired product. Instead, either a complex mixture was formed, or the starting material was recovered as such (Table 1).

**Table 1:** Conditions screened for the formation of the (±)-incarvilleatone (**1**) from (±)-**4**.

Entry	Base	Solvent	Yield (%)

1	DBU (1 equiv)	DCM	no reaction
2	DABCO (1 equiv)	dioxane/H_2_O	no reaction
3	NaH (2 equiv)	DCM	no reaction
4	Et_3_N (2 equiv)	DCM	no reaction
5	Et_3_N (2 equiv)	THF	no reaction
6	NaHMDS (2 equiv)	THF	no reaction
7	KHMDS (2 equiv)	THF	15
8	1 M aq NaOH (few drops)	THF	complex mixture

**Scheme 5:**
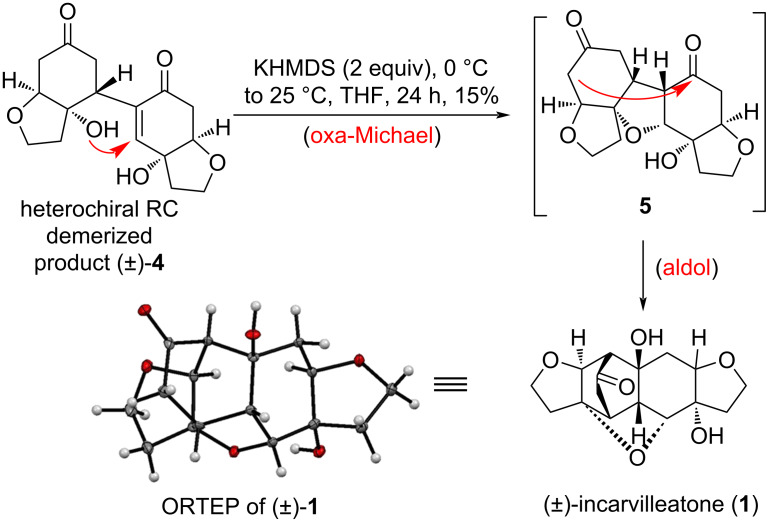
Synthesis of (±)-incarvilleatone (**1**) from RC dimerized product (±)-**4**.

However, when we treated compound (±)-**4** with a strong base such as KHMDS (2 equiv) in THF at 0 °C (Table 1, entry 7), we obtained the product as a colorless solid (15% yield) after 24 h stirring at room temperature (Scheme 5).

**Scheme 6:**
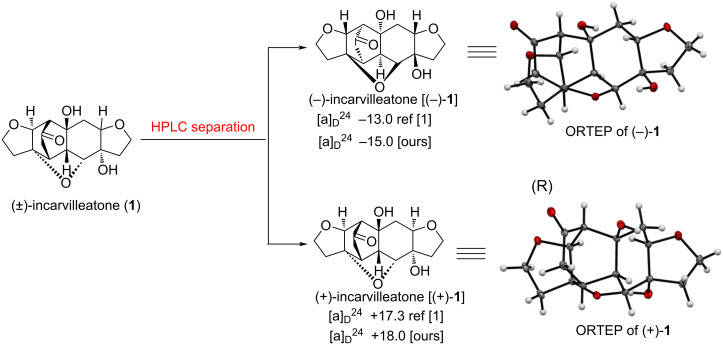
Separation of *rac*-incarvilleatone (**1**) and determination of absolute configurations of both the enantiomers using single crystal X-ray analysis.

The product was characterized as (±)-incarvilleatone (**1**) by comparison of its ^1^H NMR and ^13^C NMR spectra with the reported data [1] of natural (±)-incarvilleatone (**1**). The formation of (±)-incarvilleatone (**1**), perhaps due to RC dimerized product (±)-**4**, first undergoes oxa-Michael followed by aldol reaction in one-pot. The aldol reaction take place with intermediate **5** in basic medium due to the close proximity of the two carbonyl groups. Finally, the structure of (±)-incarvilleatone (**1**) was confirmed by single crystal X-ray analysis [16]. We then undertook separation of both the enantiomers of (±)-incarvilleatone **1** (40 mg, Scheme 6) by HPLC using a Chiralpak IA analytical column with the mobile phase MeCN/H_2_O (70:30). HPLC on a chiral stationary phase resulted in the separation of enantiomers, (−)-incarvilleatone [(−)-**1**, 15 mg] and (+)-incarvilleatone [(+)-**1**, 14 mg]. The optical rotations of the individual enantiomers were recorded and compared with the ones of Zhang and co-workers [1] reported data and were found to be nearly the same, i.e., optical rotation of −13.0 (*c* 0.30, MeOH) (isolated by Zhang and co-workers [1]) and −15.0 (*c* 0.30, MeOH) (separated by us) for (−)-incarvilleatone [(−)-**1**]; and +17.3 (*c* 0.30, MeOH) (isolated by Zhang and co-workers [1]) and + 18.0 (*c* 0.30, MeOH) (separated by us) for (+)-incarvilleatone [(+)-**1**]. We tried to crystallize both the enantiomers and after some efforts we could crystallize both the enantiomers using EtOAc as a solvent. The absolute configurations were assigned using single crystal X-ray analysis for (−)-incarvilleatone (−)-**1** as 4*R*,5*S*,8*S*,9*R*,4'*R*,5'*S*,6'*R*,7'*R*,9'*S* and for (+)-incarvilleatone [(+)-**1**] as 4*S*,5*R*,8*R*,9*S*,4'*S*,5'*R*,6'*S*,7'*S*, 9'*R* (Scheme 6).

After separation of individual enantiomers, we recorded the CD spectra of both enantiomers in MeOH. (−)-Incarvilleatone [(−)-**1**] shows a negative optical rotation and a negative Cotton effect in the CD spectrum whereas the other enantiomer (+)-incarvilleatone [(+)-**1**] showed a positive optical rotation and a positive Cotton effect in the CD spectrum (Figure S2, see Supporting Information File 1).

### Synthesis of (±)-incarviditone (**2**)

**Scheme 7:**
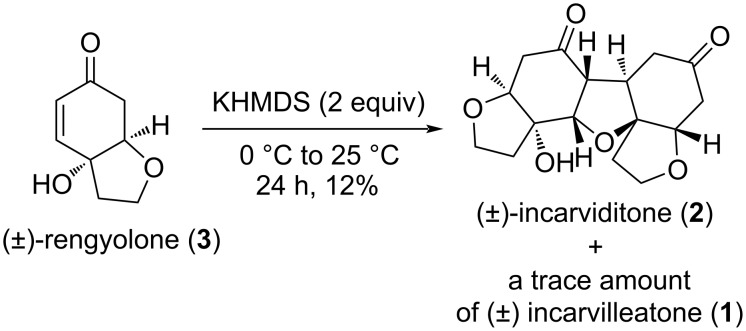
Synthesis of (±)-incarviditone (**2**).

Treatment of (±)-rengyolone (**3**) with the same base, i.e., KHMDS (2 equiv) in THF at 0 °C to rt for 24 h, resulted in the formation of a white solid (12% yield), which was identified as (±)-incarviditone (**2**) by comparison of its NMR spectra with those reported in the literature [2]. In this reaction we detected a trace amount of (±)-incarvilleatone (**1**) along with (±)-incarviditone (**2**, Scheme 7).

The mechanism of formation of (±)-incarviditone (**2**) from (±)-rengyolone (**3**) has been described by Tang et al. [4]. Overall yields of (±)-incarvilleatone (**1**) and (±)-incarviditone (**2**) from (±)-rengyolone (**3**) are 6% and 12%, respectively. Lawrence et al. [3] obtained (±)-incarvilleatone (**1**) and (±)-incarviditone (**2**) from (±)-rengyolone (**3**) in 23% and 19% yields, respectively, while Tang et al [4] furnished (±)-incarvilleatone (**1**) and (±)-incarviditone (**2**) from (±)-rengyolone (**3**) in 38% and 40% yields, respectively.

A previous study [1] showed that (±)-incarviditone (**2**) has a limited anticancer activity. The anticancer activity is closely correlated with a growth suppression. Colorimetric MTT assays are widely used to examine a growth suppression. In these assays, viable cells reduce MTT (3-(4,5-dimethylthiazol-2-yl)-2,5-diphenyltetrazolium bromide) to its insoluble formazan by oxidoreductase enzymes in a nicotinamide adenine dinucleotide phosphate (NADPH) dependent manner. Thus, reduction of MTT is closely related to the viability of the cells. Therefore, we have examined the growth-suppressive effect of all the synthesised compounds using the MTT assay [18–20].

**Figure 2:**
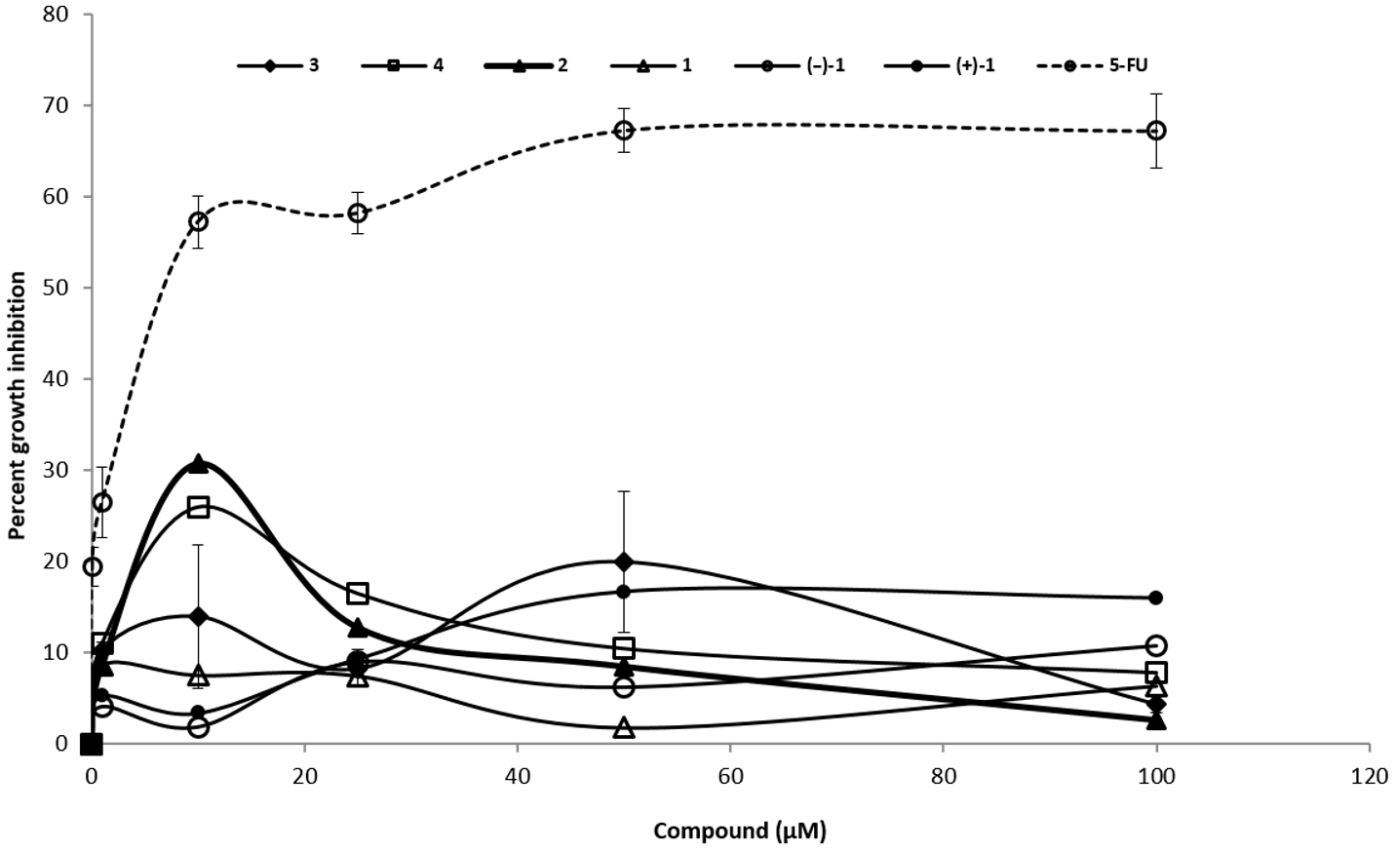
Growth suppression activity of the synthesized compounds in the breast cancer cell line MCF-7.

The results revealed that the investigated compounds have a very minimal effect on the growth of the breast cancer cell line MCF7. For instance, we did not observe the 50% growth inhibition event at 100 µM. In contrast, 5-fluorouracil potently inhibited the growth of MCF7 cells with 50% growth inhibition at 7.1 ± 0.62 µM (Figure 2).

## Conclusion

In summary, we have successfully achieved the total synthesis of (±)-incarvilleatone (**1**) starting from *rac*-rengyolone (**3**) through accelerated RC intermolecular dimerization catalyzed by TBAF to synthesize a heterochiral dimerized product (±)-**4**, followed by a one-pot oxa-Michael and aldol reaction sequence using KHMDS as a base. The synthesized (±)-incarvilleatone (**1**) was separated into its individual enantiomers by using HPLC (analytical Chiralpak IA column). The absolute configurations of both the enantiomers were determined by single crystal X-ray analysis [16]. We have also synthesized (±)-incarviditone (**2**) starting from *rac*-rengyolone (**3**) by using KHMDS as a base. The antiproliferative activity of these compounds was tested using MTT assays and the results revealed that these compounds are less efficient in inhibiting the growth of breast cancer cells.

## Supporting Information

File 1 Experimental procedures, biological protocols, ^1^H and ^13^C NMR and HRMS spectra, Figures S1 and S2, HPLC chromatograms and Tables S1–S9.*”*
