# Supplementary material for: An accelerated Rauhut–Currier dimerization enabled the synthesis of (±)-incarvilleatone and anticancer studies
Source: Beilstein J Org Chem. 2023 Feb 21;19:204–11. doi: 10.3762/bjoc.19.19 (PMC9972885; doi:10.3762/bjoc.19.19)
Supplement: File 1 — Experimental procedures, biological protocols, 1H and 13C NMR and HRMS spectra, Figures S1 and S2, HPLC chromatograms and Tables S1–S9.” [file Beilstein_J_Org_Chem-19-204-s001.pdf]

BEILSTEIN JOURNAL OF ORGANIC CHEMISTRY

Supporting Information

for

An accelerated Rauhut–Currier dimerization enabled the synthesis of (±)-incarvilleatone and anticancer studies

Tharun K. Kotammagari, Sweta Misra, Sayantan Paul, Sunita Kunte, Rajesh G. Gonnade, Manas K. Santra and Asish K. Bhattacharya

Beilstein J. Org. Chem. **2023,** *19,* 204–211. doi:10.3762/bjoc.19.19

Experimental procedures, biological protocols, ^{1}H and ^{13}C NMR and HRMS spectra, Figures S1 and S2, HPLC chromatograms and Tables S1–S9."

License and Terms: This is a supporting information file under the terms of the Creative Commons Attribution License (https://creativecommons.org/licenses/by/4.0). Please note that the reuse, redistribution and reproduction in particular requires that the author(s) and source are credited and that individual graphics may be subject to special legal provisions.
The license is subject to the *Beilstein Journal of Organic Chemistry* terms and conditions: (https://www.beilstein-journals.org/bjoc/terms)

Table of contents

1.	Experimental	S2
2.	Synthesis of (±)-incarvilleatone (**1**) and (±)-incarviditone (**2**)	S3–S4
3.	References	S5
4.	Biological protocols	S6
5.	^{1}H & ^{13}C NMR and HRMS spectra	S7–S14
6.	Figure S1 and Figure S2	S15
7.	HPLC chromatograms	S16–S18
8.	Single crystal X-ray data; Tables S1–S9	S19–S40

Experimental

All melting points were recorded on a Büchi melting point apparatus in open capillaries and are uncorrected. Commercially available reagents and dried solvents were used as received. Dry THF, MeOH and DCM were prepared following the standard procedures. Room temperature (rt) wherever mentioned corresponds to 25 °C. All dry reactions were carried out under an argon atmosphere and flash chromatography was performed with CombiFlash R_f 200i with UV/VIS and ELSD, from Isco Teledyne Inc., USA using a RediSep® column (SiO_2). ^1H NMR spectra were recorded on Bruker 500 or 400 MHz spectrometers, and ^{13}C NMR spectra were recorded at 125 or 100 MHz, respectively. Chemical shifts are reported as δ values (ppm) relative to the internal standard tetramethylsilane in $CDCl_3$. HRMS (ESI) were recorded on an Orbitrap (quadrupole plus ion trap) and TOF mass analyser. Optical rotations were recorded on a JASCO P-1020 polarimeter. HPLC was performed with Agilent HPLC system (UV detection at 200 nm, Column: Chiralpak-IA (0.46 mm X 250 mm), mobile phase: acetonitrile–water (70:30), flow rate 1.0 mL/min. X-ray intensity data measurements of compounds were carried out on a Bruker SMART APEX II CCD diffractometer with graphite-monochromatized (MoK_α= 0.71073Å) radiation at 150(2) K. The X-ray generator was operated at 50 kV and 30 mA. A preliminary set of cell constants and an orientation matrix were calculated from three sets of 36 frames. Data were collected with an ω scan width of 0.5° at different settings of φ and 2θ keeping the sample-to-detector distance fixed at 5.00 cm. The X-ray data collection was monitored by the APEX2 program (Bruker, 2006).[1] All the data were corrected for Lorentzian, polarization and absorption effects using SAINT and SADABS programs (Bruker, 2006). SHELX-97 was used for structure solution and full matrix least-squares refinement on F^2.[2] All the hydrogen atoms were placed in geometrically idealized positions and constrained to ride on their parent atoms. *ORTEP* III[3] views of both compounds were drawn with 30% probability displacement ellipsoids and H atoms are shown as small spheres of arbitrary radii.

Synthesis of ***3a,3'a-dihydroxy-3,3a,3',3'a,4,5,7,7a,7',7'a-decahydro-[4,5'-bibenzofuran]-6,6'(2H,2'H)-dione (4):***

To a solution of (±)-rengyolone (**3**, 2.6 g, 1 equiv) in THF (20 mL) at rt, TBAF in THF (1.0 M, 9.7 mL, 2 equiv) was added and the resulting solution was stirred for 24 h. Then the solution was quenched with few drops of water and the solution was concentrated in vacuo. The residue was purified by flash chromatography (CombiFlash R_f 200*i*, Isco Teledyne) using Redisep™ (silica gel, 12 g) as gradient of 1–3% of MeOH–CH_2Cl_2 to give heterochiral dimerized compound (±)-**4** (1.06 g, 41%).

3a,3'a-Dihydroxy-3,3a,3',3'a,4,5,7,7a,7',7'a-decahydro-[4,5'-bibenzofuran]-6,6'(2H,2'H)-dione (4)**:** Pale-yellow solid; m.p.: 120-123 °C; R_f 0.55 (1% MeOH-DCM);^{1}H NMR (DMSO-d_6, 400 MHz): δ_H 6.75 (s, 1H), 5.60 (s, 1H), 5.03 (s, 1H), 4.02 (t, *J*=4.9 Hz, 1H), 3.85-3.73 (m, 5H), 3.66-3.65 (m,1H), 2.79 (dd, *J*=4.3, 15.9 Hz, 1H), 2.66-2.58 (m, 3H), 2.15-2.11 (m, 2H), 1.86 (dd, *J*=3.7, 15.9 Hz 1H), 1.76-1.67(m, 3H); ^{13}C NMR (DMSO-d_6, 100 MHz): δ_C 209.4, 197.4, 148.5, 135.7, 83.4, 80.8, 77.9, 74.9, 66.0, 65.8, 42.8, 41.3, 40.6, 39.3, 37.8, 36.0; HRMS (ESI) *m/z*: calcd for $C_{16}H_{20}O_6Na$ $[M+Na]^+$ 331.1152, found 331.1150.

Synthesis of (±)-incarvilleatone (1): A stirred solution of heterochiral dihydroxy compound (±)-**4** (653 mg, 2.1 equiv) in THF (20 mL) was cooled to 0 °C, and a solution of KHMDS (399 mg, 2 equiv.) in THF was added dropwise at 0 °C slowly under argon atmosphere. The resulting reaction mixture was stirred at rt for 24 h. Then the solution was quenched with few drops of water. The resulting solution was concentrated under reduced pressure and purified by flash chromatography (CombiFlash R_f 200*i*, Isco Teledyne) using Redisep™ (silica gel, 12g) as the gradient of 1–2% of MeOH–$CHCl_3$ to furnish (±)-incarvilleatone (**1**, 101 mg, 15%). as white solid. The product was identified as (±)-incarvilleatone (**1**) by comparison of its ^{1}H NMR and ^{13}C NMR spectra with the reported spectra of natural (±)-incarvilleatone (**1**).[4]

(±)-Incarvilleatone (1)**:** White solid; R_f 0.28 (1% MeOH-$CHCl_3$); ^{1}H NMR (D_2O containing 1% CD_3OD, 400 MHz): δ_H 4.47 (d, *J* = 4.9 Hz, 1 H), 4.08-3.98 (m, 4H), 3.89-3.84 (m, 2 H), 2.91 (d, *J* = 4.3 Hz, 1H), 2.83-2.82 (m, 1H), 2.63 (dd, *J* = 3.1, 20.1 Hz, 1H), 2.56 (t, *J* = 4.3 Hz, 1H), 2.46-2.35 (m, 1H), 2.32-2.21 (m, 4H), 2.01 (ddd, *J* = 2.4, 7.3, 14.0 Hz, 1H), 1.83 (dd, *J*=9.8, 14.6 Hz, 1H); ^{13}C NMR (D_2O containing 1% CD_3OD, 100 MHz): δ_C 214.0, 88.4, 83.3, 80.9, 80.1, 79.8, 72.6, 68.8, 65.8, 59.7, 46.3 44.4, 41.8, 36.3, 33.5, 32.5; HRMS (ESI): *m/z* calcd for $C_{16}H_{20}O_6Na$ $[M+Na]^+$ 331.1152, found 331.1150.

(-)-Incarvilleatone (1): R_f 0.28 (1% MeOH- $CHCl_3$); $[\alpha]^D_{24}$ -15.0 (*c* 0.30, MeOH); ^{1}H NMR (CD_3OD, 500 MHz): δ_H 4.34 (d, *J* = 4.2 Hz, 1H), 4.01-3.97 (m, 2H), 3.95 (dd, *J*=8.8, 2.7 Hz 1H), 3.90 (dd, *J*=5.3, 9.1 Hz, 1H), 3.84-3.81 (m, 1H), 3.78 (dd, *J*=1.9, 5.0 Hz, 1H), 2.74 (d, *J*=5.0 Hz, 1H), 2.70 (dd, *J*=1.9, 3.8 Hz 1H), 2.53 (dd, *J*=3.1, 19.5 Hz, 1H), 2.45 (t, *J*=4.2 Hz, 1H), 2.34 - 2.30 (m, 1H), 2.29 (t, *J*=3.4 Hz 1H), 2.24 (d, *J*=3.4 Hz, 1 H), 2.22-2.19 (m, 2H), 2.17-2.15 (m, 1H), 1.97-1.91 (m, 1H), 1.81 (dd, *J*=9.3, 14.7 Hz, 1 H); ^{13}C NMR (CD_3OD, 125 MHz): δ_C 209.8, 88.8, 84.1, 81.8, 81.1, 79.9, 72.5, 68.6, 66.1, 60.4, 47.9, 45.7, 42.9, 37.4, 33.8, 33.6; HRMS (ESI): *m/z* calcd for $C_{16}H_{20}O_6Na$ $[M+Na]^+$ 331.1152, found 331.1152.

(+)-Incarvilleatone (1): R_f 0.28 (1% MeOH-$CHCl_3$); $[\alpha]^D_{24}$ +18.0 (*c* 0.30, MeOH); ^{1}H NMR (CD_3OD, 500 MHz): δ_H 4.34 (d, *J*=4.2 Hz, 1H), 4.01-3.97 (m, 2H), 3.95 (dd, *J*=2.7, 8.8 Hz, 1H), 3.90 (dd, *J* = 5.5, 9.3 Hz, 1H), 3.84-3.80 (m, 1H), 3.78 (dd, *J*=1.5, 5.0 Hz, 1H), 2.74 (d, *J*=5.0 Hz, 1H), 2.70 (dd, *J*=1.9, 3.8 Hz, 1H), 2.54 (dd, *J*=3.1, 19.5 Hz, 1H), 2.45 (t, *J*=4.2 Hz, 1H), 2.33-2.30 (m, 1H), 2.29 (t, *J*=3.4, 1H), 2.24 (d, *J*=3.4 Hz, 1H), 2.22 - 2.15 (m, 3H), 1.95 (td, *J*=5.2, 13.2 Hz, 1H), 1.80 (dd, *J* = 9.3, 14.7 Hz, 1H); ^{13}C NMR (CD_3OD, 125 MHz): δ_C 209.8, 88.8, 84.1, 81.8, 81.1, 79.9, 72.5, 68.6, 66.1, 60.4, 47.9, 45.7, 42.9, 37.4, 33.8, 33.6; HRMS (ESI): *m/z* calcd for $C_{16}H_{20}O_6Na$ $[M+Na]^+$ 331.1152, found 331.1150.

Synthesis of (±)-incarviditone 2: A stirred solution of (±)-rengyolone (**3**, 400 mg, 2.6 mmol) in THF (15 mL) was cooled to 0 °C, and a solution of KHMDS (1.03 g, 2 equiv) in THF (10 mL) was added dropwise at 0 °C slowly under argon atmosphere. The resulting reaction mixture was stirred at rt for 24 h. Then the solution was quenched with few drops of water. The resulting solution was concentrated under reduced pressure and purified by flash chromatography (CombiFlash R_f 200*i*, Isco Teledyne) using Redisep™ (silica gel, 12g) as the gradient of 0.5–1% of MeOH-$CHCl_3$ to give (±)-incarviditone (**2**, 48 mg, 12%) as colorless liquid. The product was identified as (±)-incarviditone (**2**) by comparison of its ^{1}H NMR and ^{13}C NMR spectra with the reported spectra of natural (±)-incarviditone (**2**).[5]

***(±)-Incarviditone (2)*:** Colorless liquid; R_f 0.48 (1% MeOH-$CHCl_3$); ^{1}H NMR (CD_3OD, 400 MHz): δ_H 4.58 (d, *J*=7.3 Hz, 1H), 4.07 (t, *J* = 4.9 Hz 1H), 4.02-3.96 (m, 4H), 3.94-3.90 (m, 1H), 2.97-2.93 (m, 1H), 2.89 (t, *J*=7.9 Hz, 1H), 2.85-2.81 (m, 1H), 2.65 (dd, *J*=4.3, 17.8 Hz 1H), 2.59 (d, *J*=5.5 Hz, 1H), 2.54-2.49 (m, 2H), 2.42-2.37 (m, 1H), 2.33-2.27 (m, 2H), 1.98 (ddd, *J* = 5.5, 7.3, 12.8 Hz, 1H); ^{13}C NMR (CD_3OD, 100 MHz): δ_C 211.1, 209.3, 90.3, 83.2, 82.7, 81.9, 79.3, 67.6, 67.3, 55.7, 45.2, 43.8, 43.3, 40.4, 39.4, 37.9; HRMS (ESI): *m/z* calcd for $C_{16}H_{20}O_6Na$ $[M+Na]^+$ 331.1152, found 331.1152.

References:

1. Bruker (**2006**). *APEX2*, *SAINT* and *SADABS*. Bruker AXS Inc., Madison, Wisconsin, USA.

2. Sheldrick, G. M. *Acta Crystallogr*. **2008**, *A64*, 112.

3. Farrugia, L. J. *J. Appl. Cryst.* **1997**, *30*, 565–565.

4. Gao, Y. P.; Shen, Y. H.; Zhang, S. D.; Tian, J. M.; Zeng, H. W.; Ye, J.; Li, H. L.; Shan, L.; Zhang, W. D. *Org. Lett*. **2012**, *14*, 1954-1957.

5. Chen, Y. Q.; Shen, Y. H.; Su, Y. Q.; Kong, L. Y.; Zhang, W. D. *Chem. Biodiversity*. **2009**, *6*, 779-783.

Biological protocols

Anticancer assay:

Cell culture

Breast cancer MCF7 cells were grown in DMEM media (GIBCO). MCF7 cell line was a kind gift from Michael R. Green (UMass Medical School, USA). Cells were grown in media supplemented with 10% fetal bovine serum (Gibco) at 37 °C in 5% CO_2 under humidified conditions.

Cell survival assays

The cytotoxic effect of the compounds on MCF7 cells was determined after three independent experiments using standard 3-(4,5-dimethylthiazol-2-yl)-2,5-diphenyltetrazolium bromide (MTT) assays. Five thousand cells were seeded in each well of a 96-welled plate. At 24 h of post seeding, cells were treated with different concentrations of the compounds (0–100 μM). Vehicle (DMSO) treated cells were used as control. After 48 h of treatment, cells were grown in the presence of 0.83 mg/mL MTT reagent for additional 4 h. The media containing the MTT reagent was then replaced with 100 μL/well of MTT solvent (5 mM HCl and 0.1% Triton X-100 in isopropanol) and incubated at 25 °C for 10 min with gentle shaking and subsequently absorbance was taken at 575 nm in Thermo Scientific Multiskan Go plate reader. The numbers of live cells after 48 h of treatment were calculated based on the readout of reduction of the MTT salt into its formazan derivative, which has an absorbance at 575 nm. Growth of vehicle treated cells was taken as 100%. An average of three experiments was plotted as percentage of growth inhibition.

^{1}H & ^{13}C NMR and HRMS Spectra

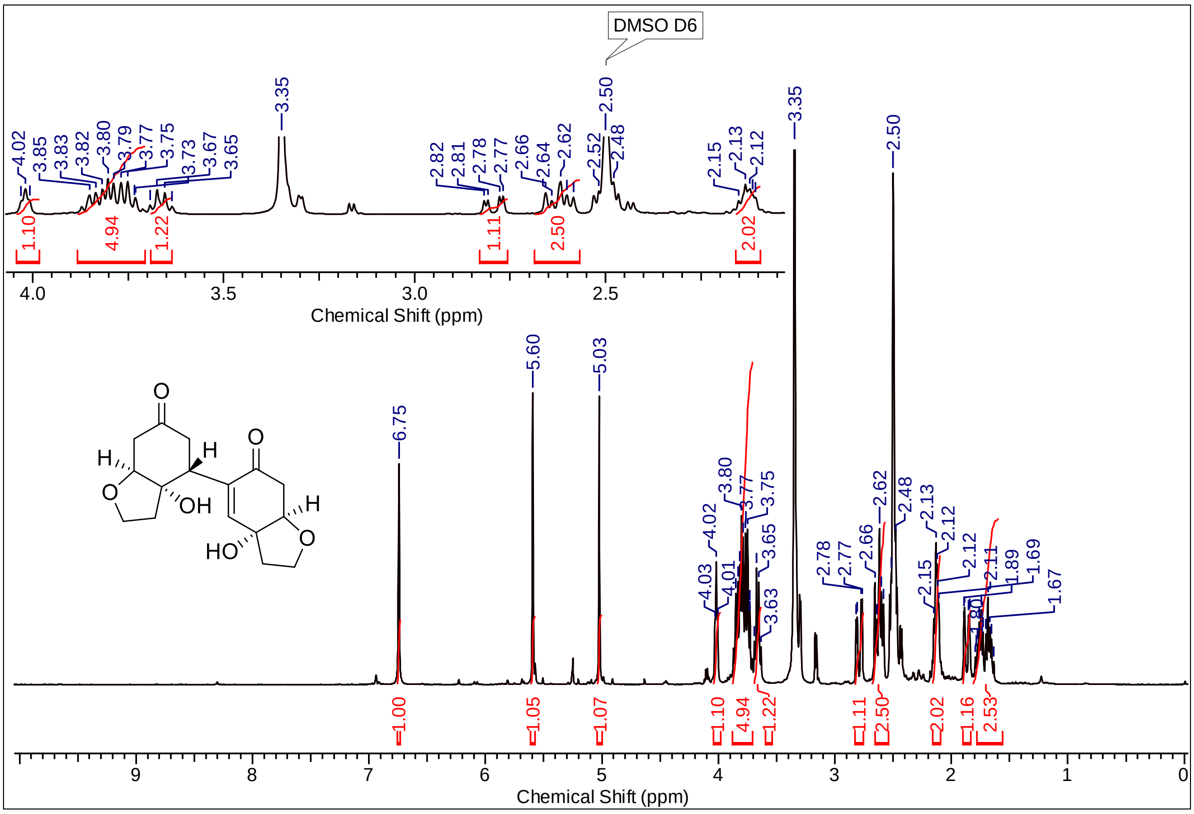

^{1}H NMR (DMSO-d_6, 400 MHz) of compound (±)-4.

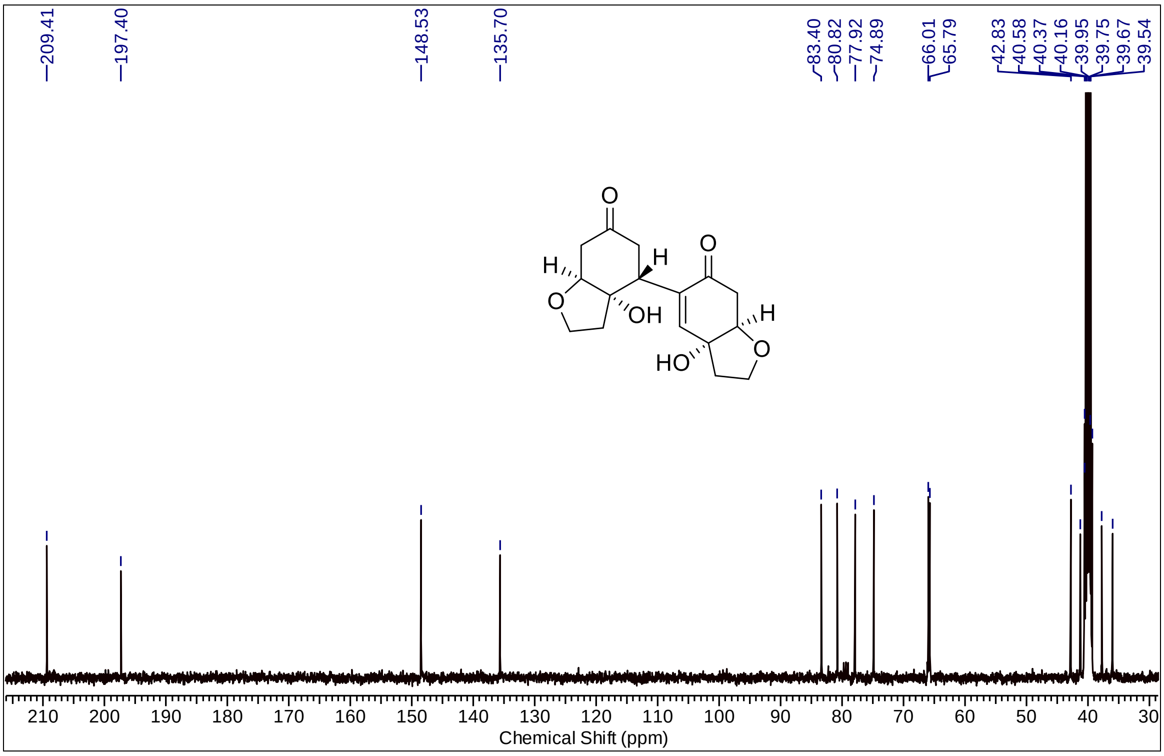

^{13}C NMR (DMSO-d_6, 100 MHz) of compound 4.

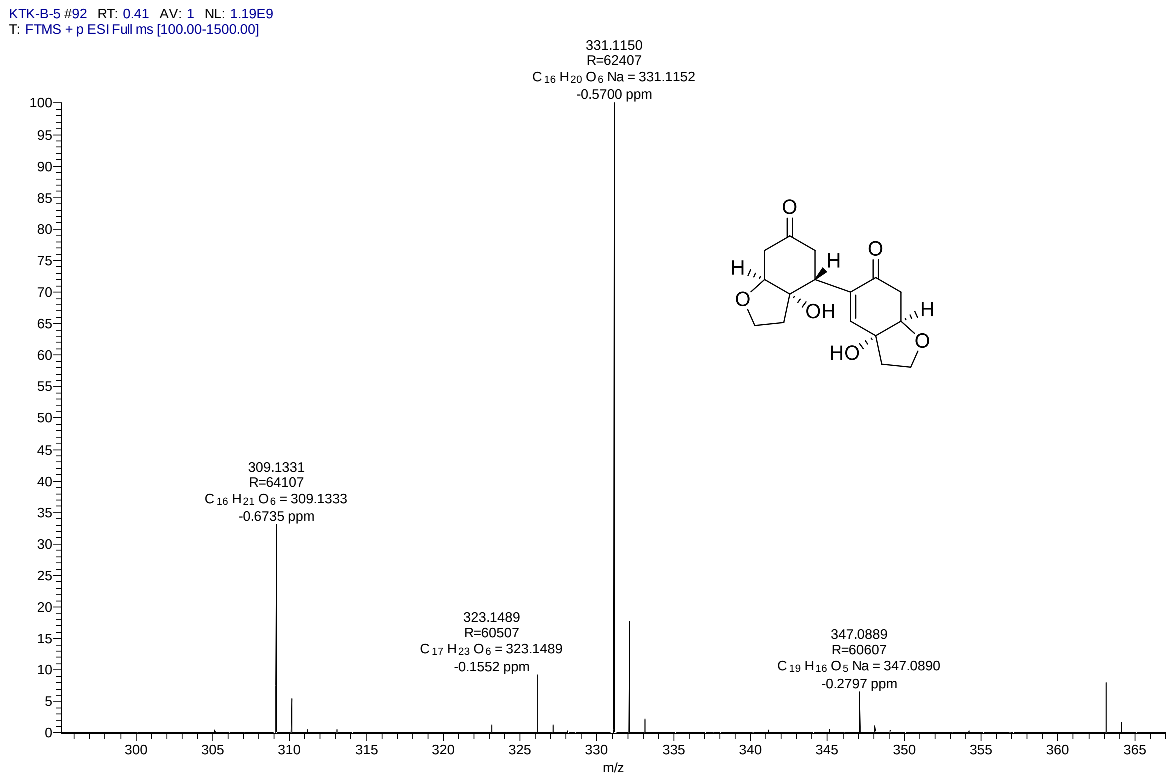

HRMS of compound 4

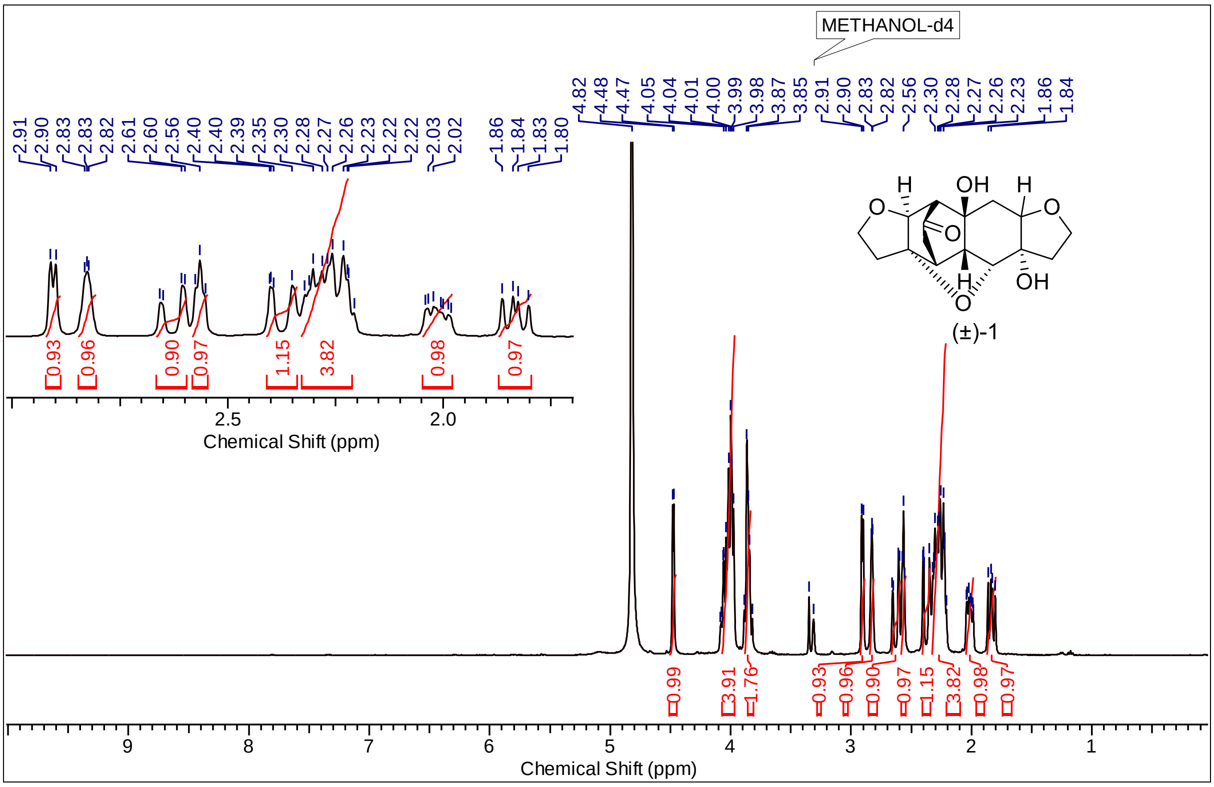

^{1}H NMR (400 MHz, D_2O containing 1% CD_3OD) of compound 1.

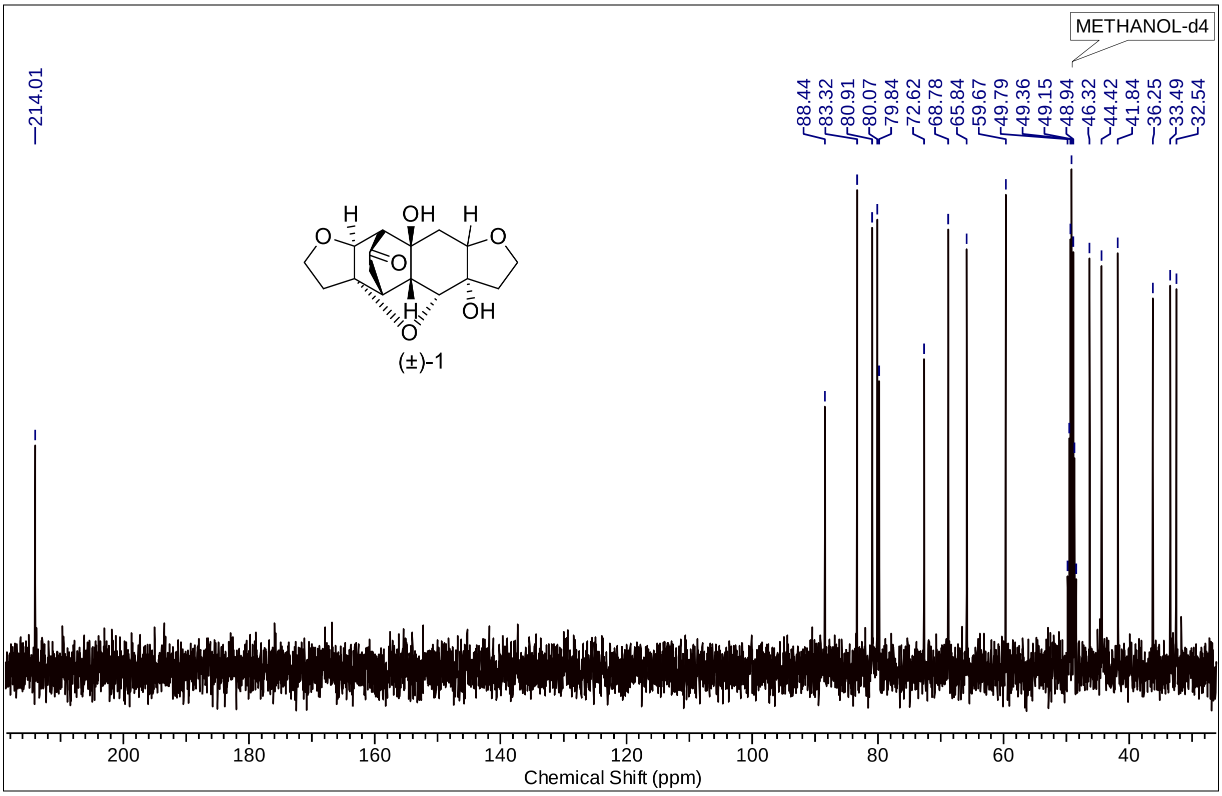

^{13}C NMR (100 MHz, D_2O containing 1% CD_3OD) of compound 1.

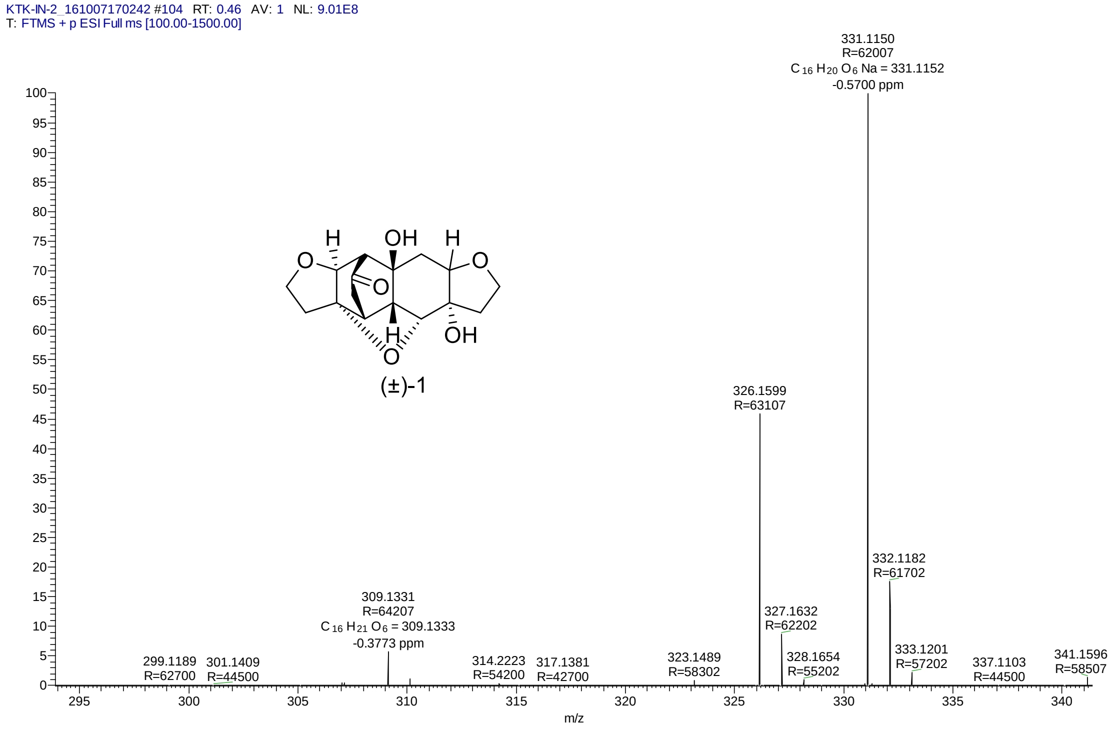

HRMS of compound 1.

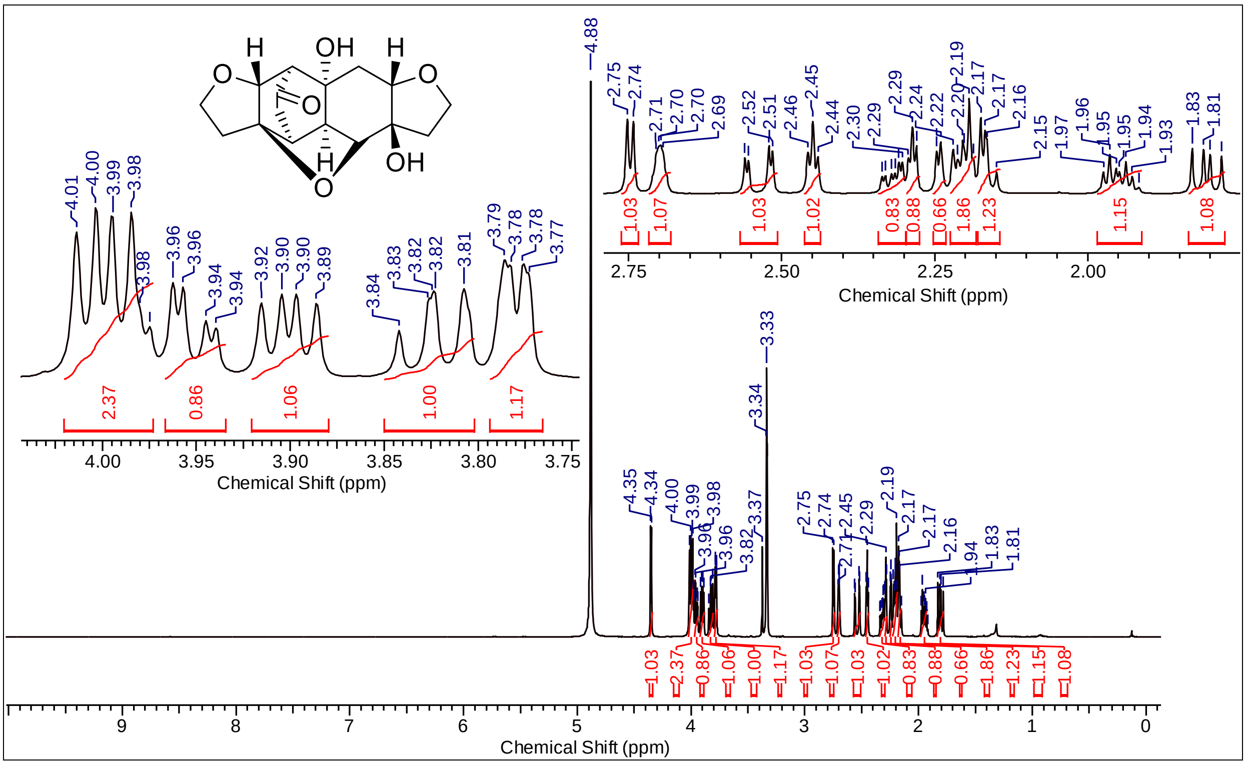

^{1}H NMR (500 MHz, CD3OD) of compound (−)-1.

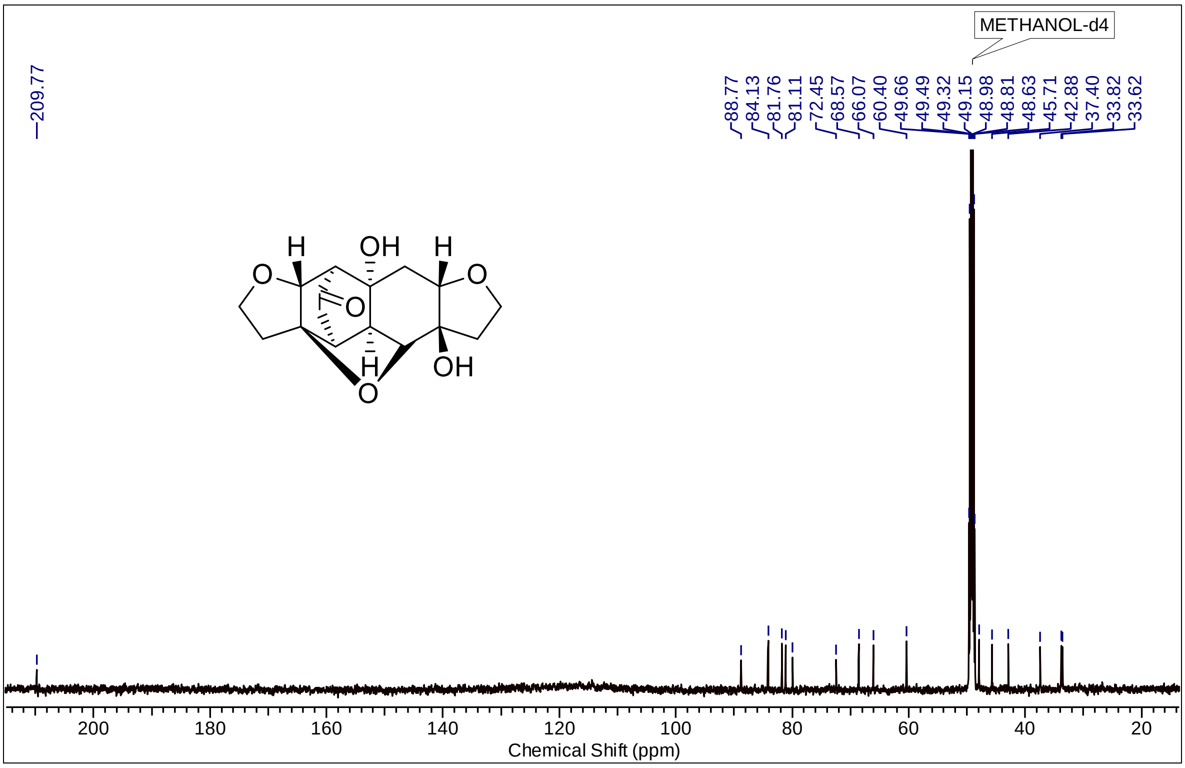

^{13}C NMR (125 MHz, CD3OD) of compound (−)-1.

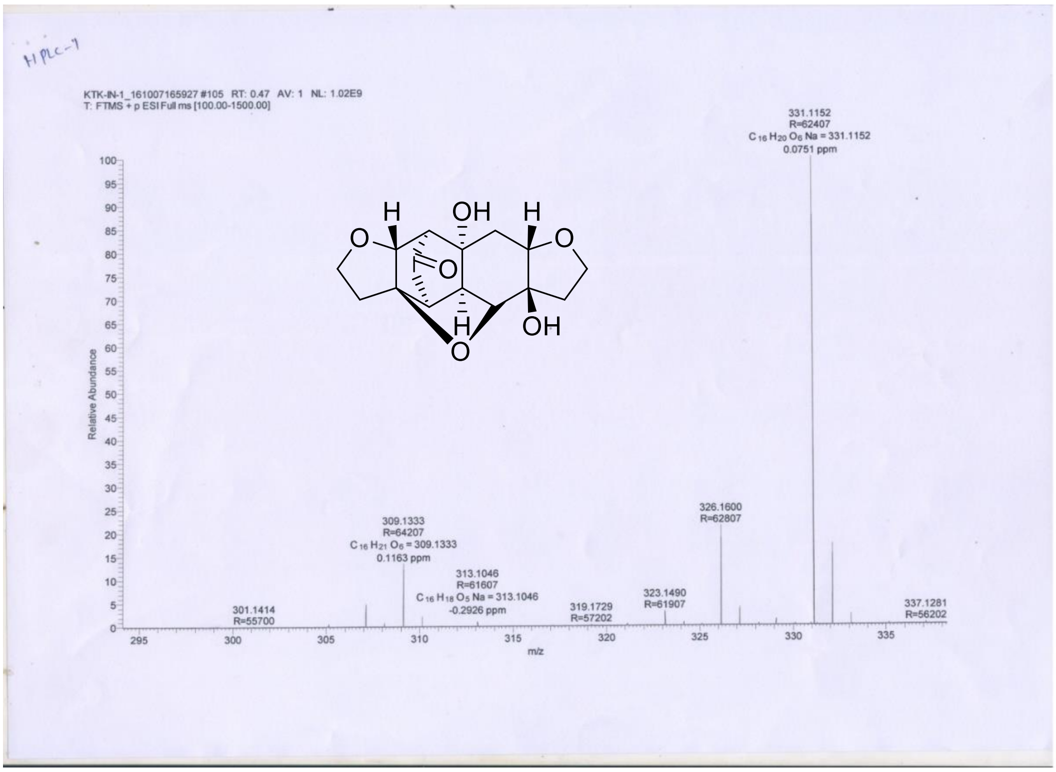

HRMS of compound (−)-1.

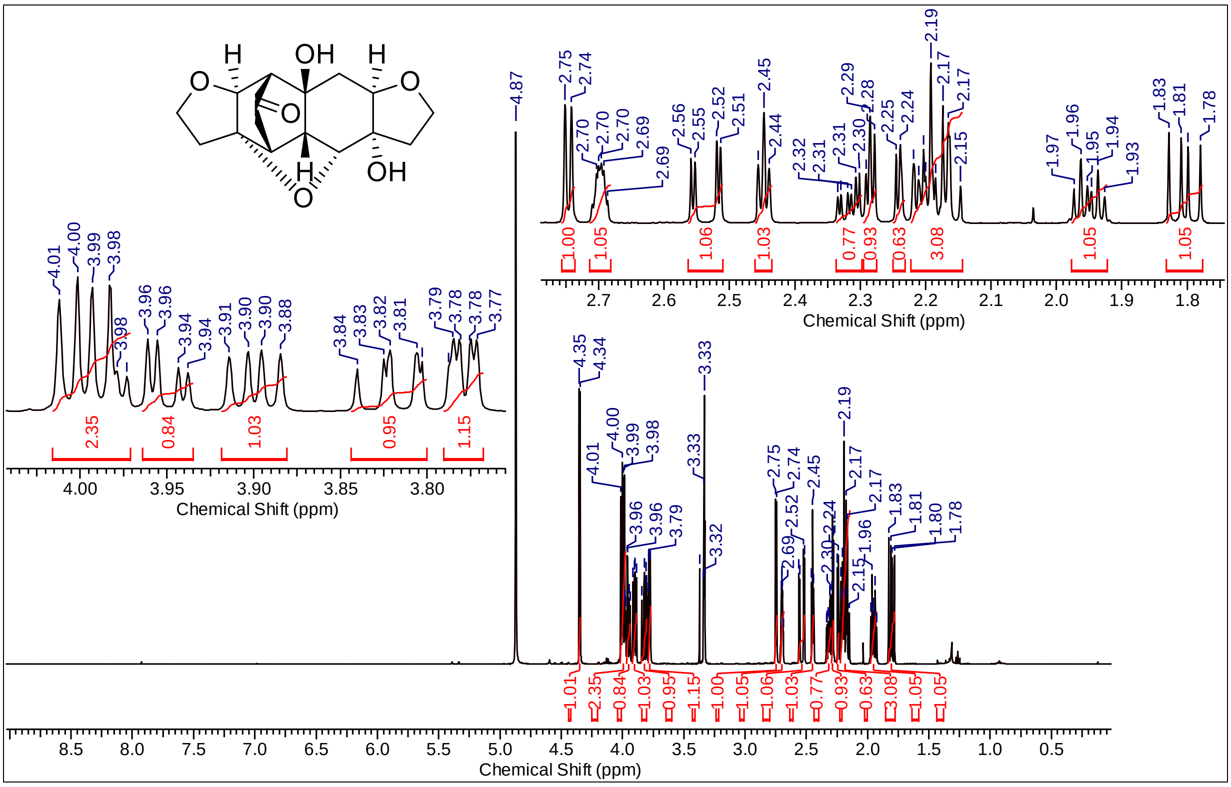

^{1}H NMR (500 MHz, CD3OD) of compound (+)-1.

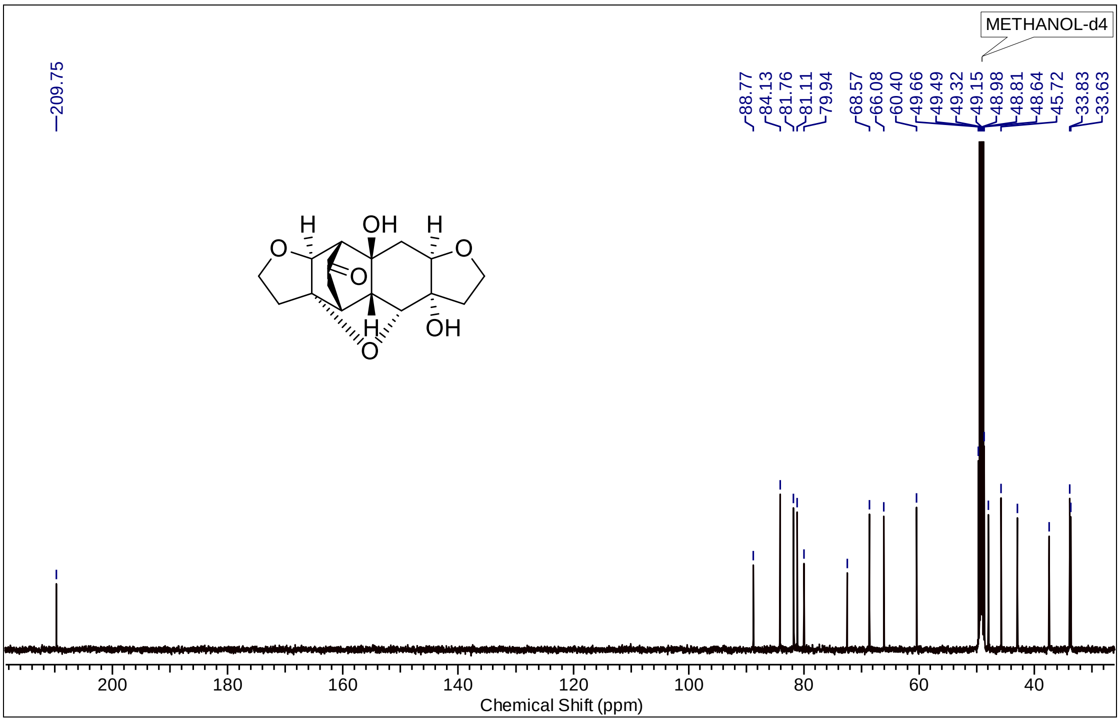

^{13}C NMR (125 MHz, CD3OD) of compound (+)-1.

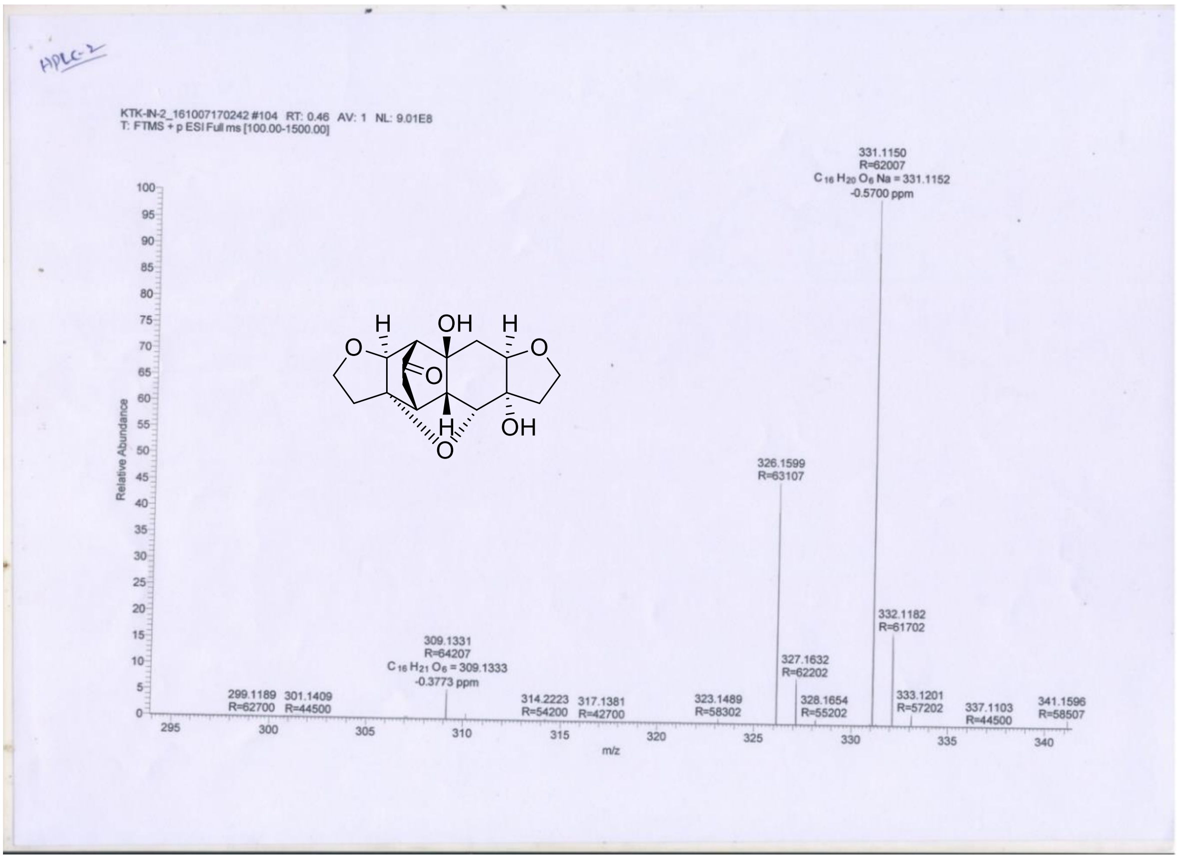

HRMS of compound (+)-1.

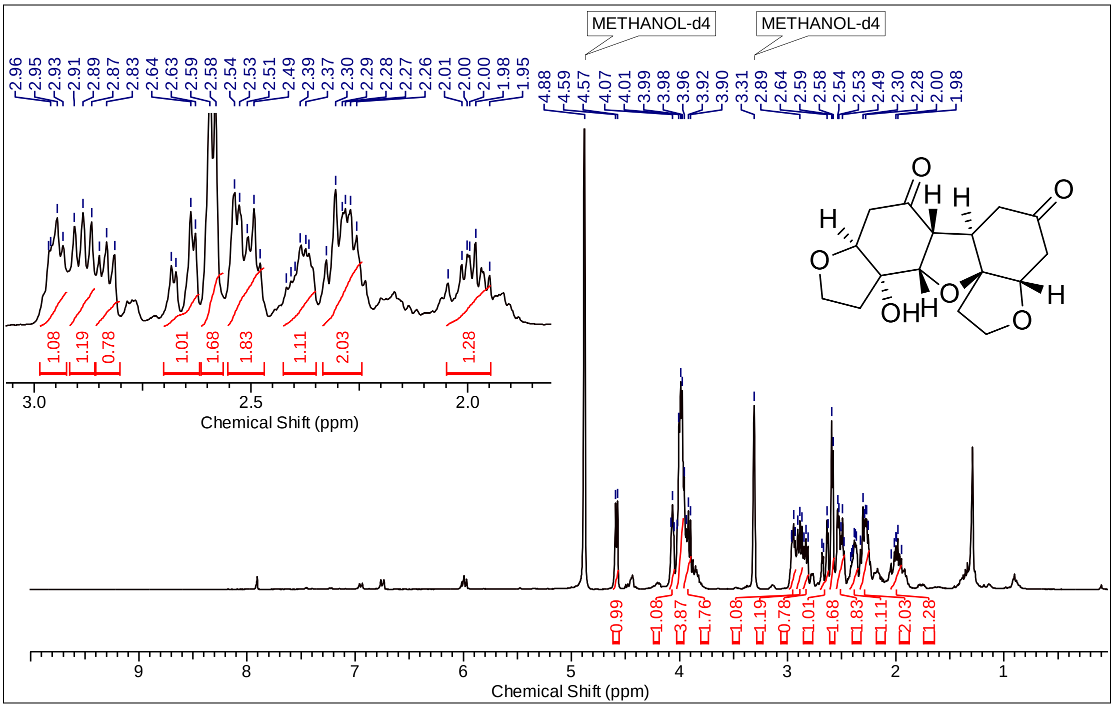

^{1}H NMR (400 MHz, CD3OD) of compound 2.

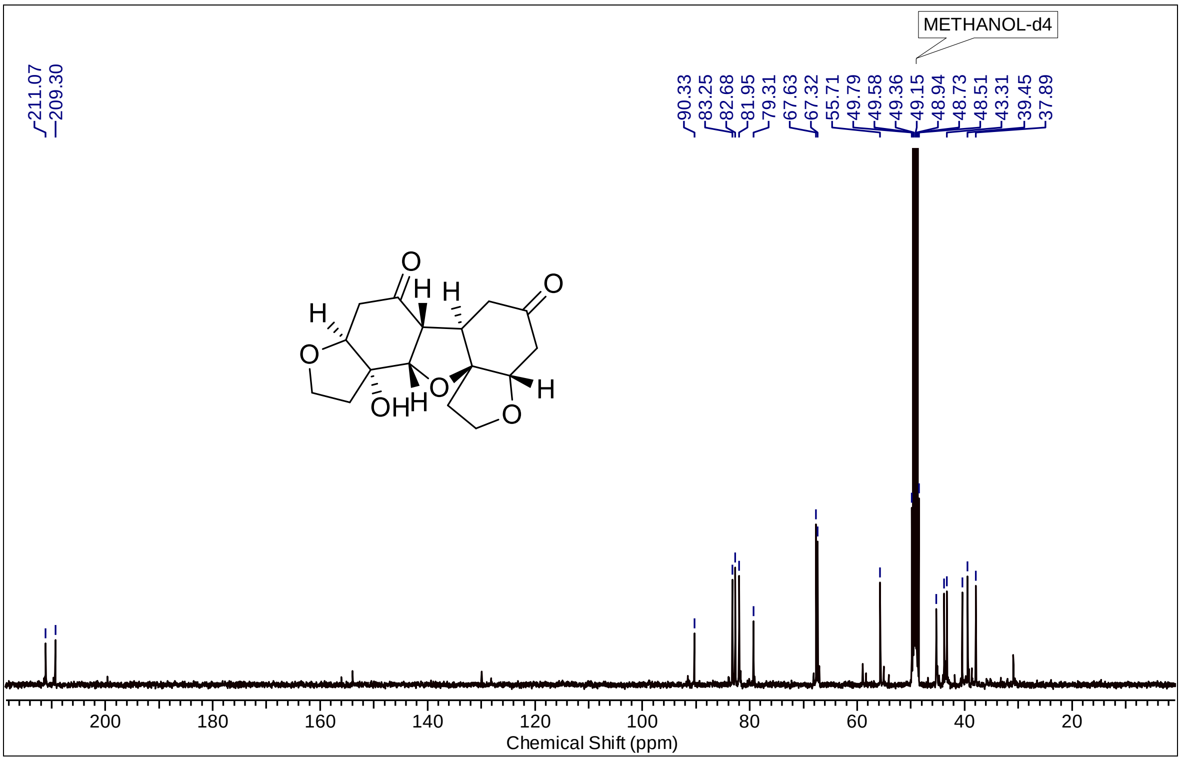

^{13}C NMR (100 MHz, CD3OD) of compound 2.

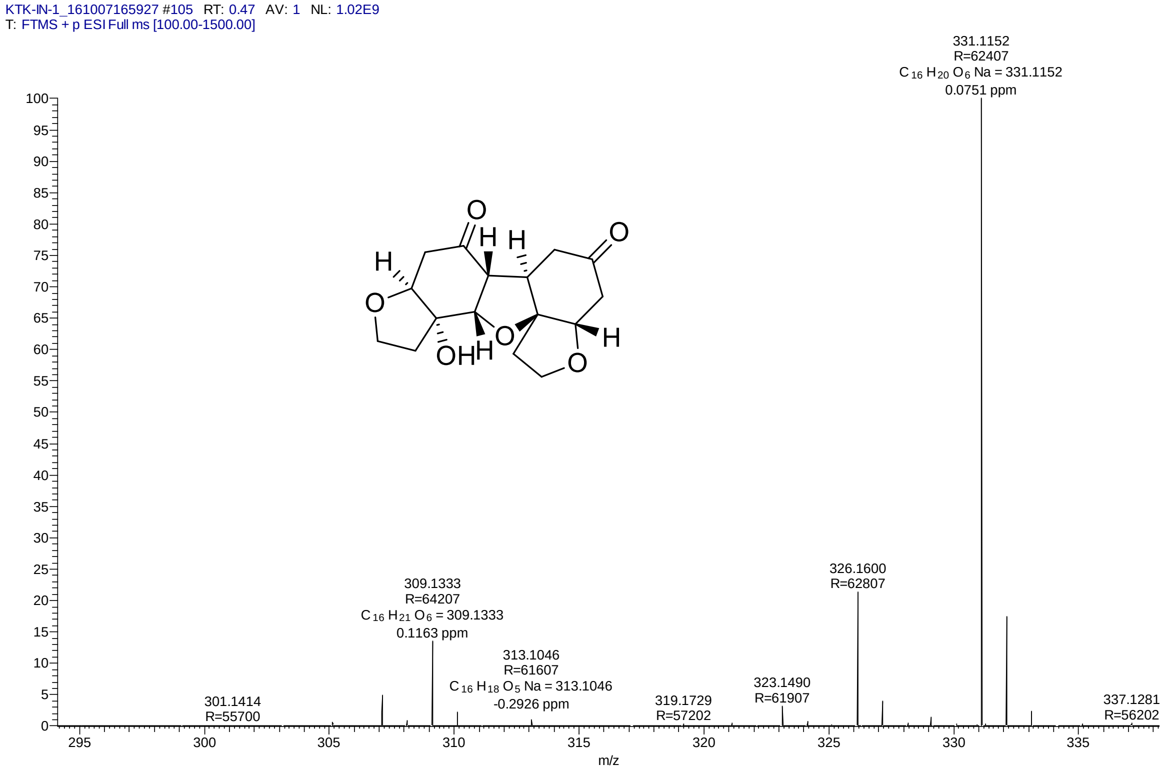

HRMS of compound 2.

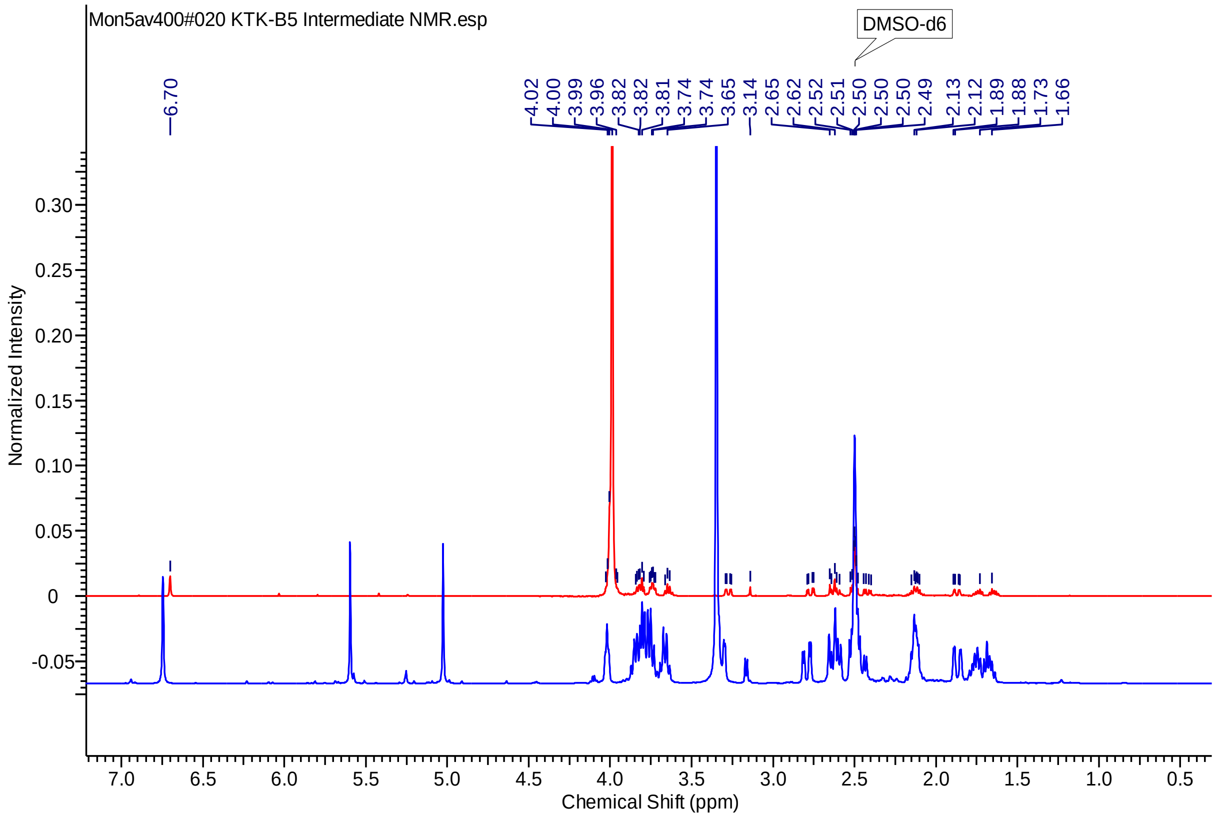

Figure S1. D_2O shake experiment: ^{1}H NMR of the compound **4** with D_2O (red), ^{1}H NMR of the compound without D_2O (blue).

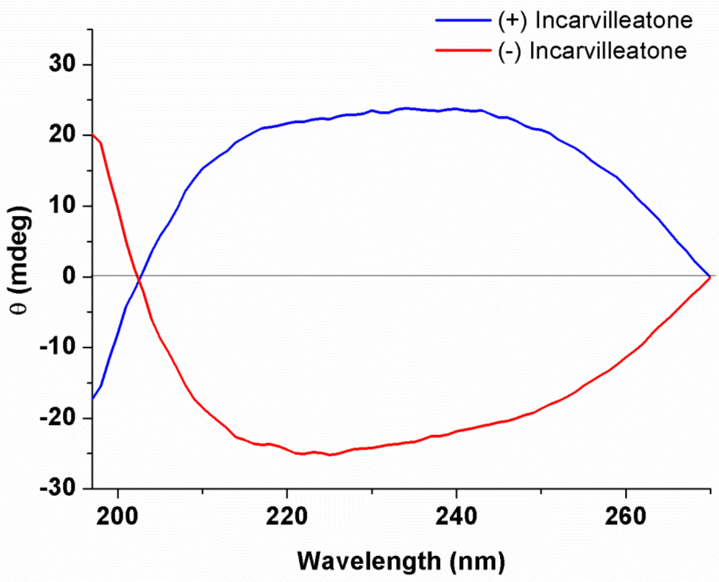

Figure S2. Circular dichroism (CD) spectra of the (−)-incarvilleatone (**1**) and (+)-incarvilleatone (**1**).

HPLC Chromatograms

D-7000 HPLC System Manager Report

Analyzed: 11/15/16 11:44 AM

Reported: 11/15/16 12:03 PM
Processed: 11/15/16 12:03 PM

Data Path: C:\WIN32APP\HSM\HPLC\DATA\9115\
Processing Method:.cal

System(acquisition): Sys 1
Application: HPLC
Sample Name: KTK-IN-2
Injection from this vial: 1 of 1
Sample Description: ACN:H2O(70:30)

Series:9115
Volume: 10.0 ul

Chrom Type: HPLC Channel : 1

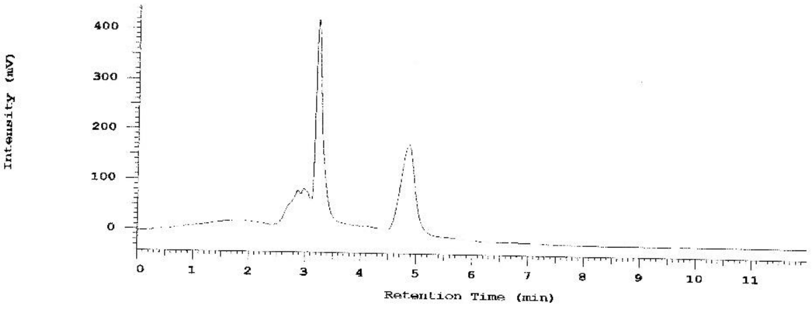

No.	RT	Area	Conc 1	BC
1	3.21	2947499	49.302	BB
2	4.85	3030915	50.698	BB
		5978414	100.000	

Peak rejection level: 0

```
Project Leader: Dr. A. K. Bhattacharya
Column      : CHIRALPAK IA, (250 mmx4.6mm),5um
Mobile Ph   : Acetonitrile :WATER(70:30)
Wavelength  : 200nm
Flow        : 1.0ml/min.
Inject vol: 15ul
```

HPLC report of (±)-incarvilleatone (1).

D-7000 HPLC System Manager Report

Analyzed: 11/24/16 04:49 PM

Reported: 11/24/16 04:59 PM
Processed: 11/24/16 04:59 PM

Data Path: C:\WIN32APP\HSM\HPLC\DATA\9145\
Processing Method: cal
System(acquisition): Sys 1
Application: HPLC
Sample Name: KTK-IN-2 (1)
Injection from this vial: 1 of 1
Sample Description: ACN:H2O(70:30)

Series:9145
Volume: 10.0 ul

Chrom Type: HPLC Channel : 1

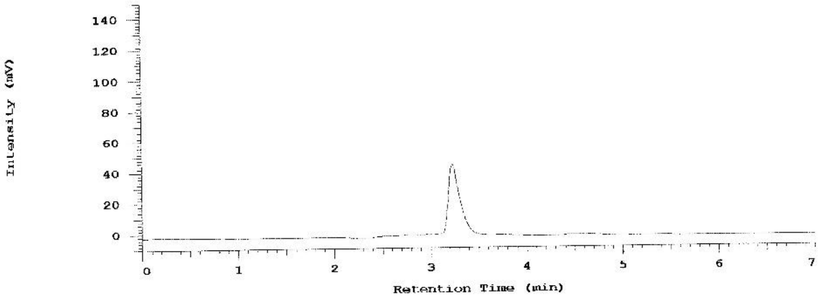

No.	RT	Area	Conc 1	BC
1	3.23	371858	100.000	BB
		371858	100.000	

Peak rejection level: 0

```
Project Leader: Dr.A. K. BHATTACHARYA
Column      : CHIRALPAK IA, (250 mmx4.6mm),5um
Mobile Ph   : ACN:H2O(70:30)
Wavelength  : 200nm
Flow        : 1.0ml/min.
Inject vol: 20ul
```

HPLC report of (−)-1.

D-7000 HPLC System Manager Report

Analyzed: 11/24/16 04:56 PM

Reported: 11/24/16 05:06 PM
Processed: 11/24/16 05:06 PM

Data Path: C:\WIN32APP\HSM\HPLC\DATA\9146\
Processing Method: cal

System(acquisition): Sys 1
Application: HPLC
Sample Name: KTK-IN-2 (2)
Injection from this vial: 1 of 1
Sample Description: ACN:H2O(70:30)

Series:9146
Volume: 10.0 ul

Chrom Type: HPLC Channel : 1

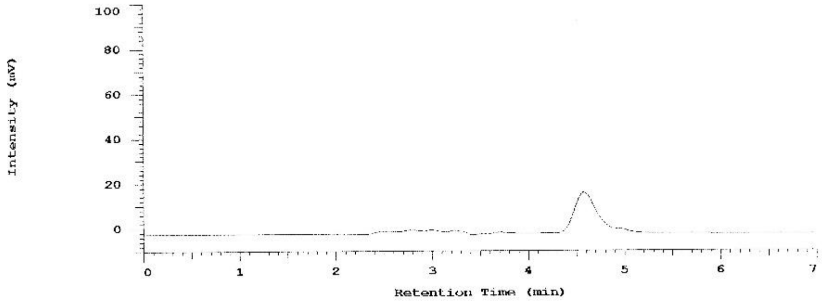

No.	RT	Area	Conc 1	BC
1	4.58	252124	100.000	BB
		252124	100.000	

Peak rejection level: 0

```
Project Leader: Dr.A. K. BHATTACHARYA
Column      : CHIRALPAK IA, (250 mmx4.6mm),5um
Mobile Ph   : ACN:H2O(70:30)
Wavelength  : 200nm
Flow        : 1.0ml/min.
Inject vol: 20ul
```

HPLC report of (+)-1.

Single crystal X-ray data

Table S1. Crystal data and structure refinement for (±)-incarvilleatone (**1**).

Identification code	(±)-incarvilleatone (**1**)	
Empirical formula	C_{16} H_{20} O_6	
Formula weight	308.32	
Temperature	150(2) K	
Wavelength	0.71073 Å	
Crystal system	Monoclinic	
Space group	$P2_1/c$	
Unit cell dimensions	a = 12.3496(17) Å	α = 90°.
	b = 9.3302(13) Å	β = 96.139(2)°.
	c = 12.0164(17) Å	γ = 90°.
Volume	1376.6(3) Å^3	
Z	4	
Density (calculated)	1.488 Mg/m^3	
Absorption coefficient	0.114 mm^{-1}	
F(000)	656	
Crystal size	0.240 x 0.180 x 0.120 mm^3	
Theta range for data collection	2.742 to 27.998°.	
Index ranges	-14<=h<=16, -12<=k<=12, -15<=l<=14	
Reflections collected	13304	
Independent reflections	3325 [R(int) = 0.0691]	
Completeness to theta = 25.242°	99.9 %	
Absorption correction	Semi-empirical from equivalents	
Max. and min. transmission	0.986 and 0.973	
Refinement method	Full-matrix least-squares on F^2	
Data / restraints / parameters	3325 / 0 / 201	
Goodness-of-fit on F^2	1.100	
Final R indices [I>2sigma(I)]	R1 = 0.0644, wR2 = 0.1275	
R indices (all data)	R1 = 0.0923, wR2 = 0.1387	
Extinction coefficient	n/a	
Largest diff. peak and hole	0.385 and -0.259 e.Å^{-3}	

Table S2. Bond lengths [Å] and angles [°] for (±)-incarvilleatone (**1**).

O(1)-C(9)	1.424(3)
O(1)-C(2)	1.441(3)
C(2)-C(3)	1.526(3)
C(2)-H(2A)	0.9900
C(2)-H(2B)	0.9900
O(2)-C(7)	1.219(3)
C(3)-C(4)	1.536(3)
C(3)-H(3A)	0.9900
C(3)-H(3B)	0.9900
O(3)-C(4)	1.434(3)
O(3)-C(5')	1.458(3)
C(4)-C(5)	1.527(3)
C(4)-C(9)	1.554(3)
C(5)-C(6)	1.532(3)
C(5)-C(6')	1.544(3)
C(5)-H(5)	1.0000
C(6)-C(7)	1.505(3)
C(6)-H(6A)	0.9900
C(6)-H(6B)	0.9900
C(7)-C(8)	1.513(3)
C(8)-C(9)	1.530(3)
C(8)-C(7')	1.559(3)
C(8)-H(8)	1.0000
C(9)-H(9)	1.0000
O(1')-C(9')	1.441(3)
O(1')-C(2')	1.447(3)
C(2')-C(3')	1.541(3)
C(2')-H(2'A)	0.9900
C(2')-H(2'B)	0.9900
C(3')-C(4')	1.526(3)
C(3')-H(3'A)	0.9900
C(3')-H(3'B)	0.9900
C(4')-O(4')	1.431(3)
C(4')-C(5')	1.526(3)
C(4')-C(9')	1.528(3)
O(4')-H(4'A)	0.8400

C(5')-C(6')	1.542(3)
C(5')-H(5')	1.0000
C(6')-C(7')	1.546(3)
C(6')-H(6')	1.0000
C(7')-O(7')	1.443(3)
C(7')-C(8')	1.528(3)
O(7')-H(7'A)	0.8400
C(8')-C(9')	1.524(3)
C(8')-H(8'A)	0.9900
C(8')-H(8'B)	0.9900
C(9')-H(9')	1.0000
C(9)-O(1)-C(2)	104.37(17)
O(1)-C(2)-C(3)	104.84(18)
O(1)-C(2)-H(2A)	110.8
C(3)-C(2)-H(2A)	110.8
O(1)-C(2)-H(2B)	110.8
C(3)-C(2)-H(2B)	110.8
H(2A)-C(2)-H(2B)	108.9
C(2)-C(3)-C(4)	103.55(19)
C(2)-C(3)-H(3A)	111.1
C(4)-C(3)-H(3A)	111.1
C(2)-C(3)-H(3B)	111.1
C(4)-C(3)-H(3B)	111.1
H(3A)-C(3)-H(3B)	109.0
C(4)-O(3)-C(5')	108.00(16)
O(3)-C(4)-C(5)	103.96(17)
O(3)-C(4)-C(3)	111.64(18)
C(5)-C(4)-C(3)	118.67(19)
O(3)-C(4)-C(9)	109.63(18)
C(5)-C(4)-C(9)	109.42(18)
C(3)-C(4)-C(9)	103.44(18)
C(4)-C(5)-C(6)	112.59(19)
C(4)-C(5)-C(6')	98.11(17)
C(6)-C(5)-C(6')	114.58(18)
C(4)-C(5)-H(5)	110.3
C(6)-C(5)-H(5)	110.3
C(6')-C(5)-H(5)	110.3

C(7)-C(6)-C(5)	109.94(18)
C(7)-C(6)-H(6A)	109.7
C(5)-C(6)-H(6A)	109.7
C(7)-C(6)-H(6B)	109.7
C(5)-C(6)-H(6B)	109.7
H(6A)-C(6)-H(6B)	108.2
O(2)-C(7)-C(6)	123.4(2)
O(2)-C(7)-C(8)	123.9(2)
C(6)-C(7)-C(8)	112.71(19)
C(7)-C(8)-C(9)	106.10(18)
C(7)-C(8)-C(7')	104.89(17)
C(9)-C(8)-C(7')	111.59(17)
C(7)-C(8)-H(8)	111.3
C(9)-C(8)-H(8)	111.3
C(7')-C(8)-H(8)	111.3
O(1)-C(9)-C(8)	109.67(17)
O(1)-C(9)-C(4)	106.15(17)
C(8)-C(9)-C(4)	110.68(18)
O(1)-C(9)-H(9)	110.1
C(8)-C(9)-H(9)	110.1
C(4)-C(9)-H(9)	110.1
C(9')-O(1')-C(2')	107.94(16)
O(1')-C(2')-C(3')	107.13(17)
O(1')-C(2')-H(2'A)	110.3
C(3')-C(2')-H(2'A)	110.3
O(1')-C(2')-H(2'B)	110.3
C(3')-C(2')-H(2'B)	110.3
H(2'A)-C(2')-H(2'B)	108.5
C(4')-C(3')-C(2')	103.12(18)
C(4')-C(3')-H(3'A)	111.1
C(2')-C(3')-H(3'A)	111.1
C(4')-C(3')-H(3'B)	111.1
C(2')-C(3')-H(3'B)	111.1
H(3'A)-C(3')-H(3'B)	109.1
O(4')-C(4')-C(5')	109.35(18)
O(4')-C(4')-C(3')	105.48(18)
C(5')-C(4')-C(3')	115.32(18)
O(4')-C(4')-C(9')	110.28(18)

C(5')-C(4')-C(9')	114.42(18)
C(3')-C(4')-C(9')	101.43(18)
C(4')-O(4')-H(4'A)	109.5
O(3)-C(5')-C(4')	107.58(17)
O(3)-C(5')-C(6')	105.64(17)
C(4')-C(5')-C(6')	118.37(18)
O(3)-C(5')-H(5')	108.3
C(4')-C(5')-H(5')	108.3
C(6')-C(5')-H(5')	108.3
C(5')-C(6')-C(5)	100.64(17)
C(5')-C(6')-C(7')	115.51(18)
C(5)-C(6')-C(7')	108.40(17)
C(5')-C(6')-H(6')	110.6
C(5)-C(6')-H(6')	110.6
C(7')-C(6')-H(6')	110.6
O(7')-C(7')-C(8')	108.00(17)
O(7')-C(7')-C(6')	105.48(17)
C(8')-C(7')-C(6')	113.05(18)
O(7')-C(7')-C(8)	106.83(17)
C(8')-C(7')-C(8)	113.30(18)
C(6')-C(7')-C(8)	109.67(17)
C(7')-O(7')-H(7'A)	109.5
C(9')-C(8')-C(7')	115.68(18)
C(9')-C(8')-H(8'A)	108.4
C(7')-C(8')-H(8'A)	108.4
C(9')-C(8')-H(8'B)	108.4
C(7')-C(8')-H(8'B)	108.4
H(8'A)-C(8')-H(8'B)	107.4
O(1')-C(9')-C(8')	109.77(17)
O(1')-C(9')-C(4')	104.17(17)
C(8')-C(9')-C(4')	111.53(18)
O(1')-C(9')-H(9')	110.4
C(8')-C(9')-H(9')	110.4
C(4')-C(9')-H(9')	110.4

Symmetry transformations used to generate equivalent atoms:

Table S3. Torsion angles [°] for (±)-incarvilleatone (**1**).

C(9)-O(1)-C(2)-C(3)	43.1(2)
O(1)-C(2)-C(3)-C(4)	-30.6(2)
C(5')-O(3)-C(4)-C(5)	-30.3(2)
C(5')-O(3)-C(4)-C(3)	-159.34(18)
C(5')-O(3)-C(4)-C(9)	86.6(2)
C(2)-C(3)-C(4)-O(3)	-110.0(2)
C(2)-C(3)-C(4)-C(5)	129.1(2)
C(2)-C(3)-C(4)-C(9)	7.8(2)
O(3)-C(4)-C(5)-C(6)	167.45(17)
C(3)-C(4)-C(5)-C(6)	-67.9(3)
C(9)-C(4)-C(5)-C(6)	50.4(2)
O(3)-C(4)-C(5)-C(6')	46.5(2)
C(3)-C(4)-C(5)-C(6')	171.18(19)
C(9)-C(4)-C(5)-C(6')	-70.6(2)
C(4)-C(5)-C(6)-C(7)	-54.8(2)
C(6')-C(5)-C(6)-C(7)	56.2(2)
C(5)-C(6)-C(7)-O(2)	176.9(2)
C(5)-C(6)-C(7)-C(8)	-2.6(3)
O(2)-C(7)-C(8)-C(9)	-118.9(2)
C(6)-C(7)-C(8)-C(9)	60.7(2)
O(2)-C(7)-C(8)-C(7')	122.8(2)
C(6)-C(7)-C(8)-C(7')	-57.6(2)
C(2)-O(1)-C(9)-C(8)	-157.36(18)
C(2)-O(1)-C(9)-C(4)	-37.8(2)
C(7)-C(8)-C(9)-O(1)	52.9(2)
C(7')-C(8)-C(9)-O(1)	166.56(17)
C(7)-C(8)-C(9)-C(4)	-63.9(2)
C(7')-C(8)-C(9)-C(4)	49.8(2)
O(3)-C(4)-C(9)-O(1)	136.91(17)
C(5)-C(4)-C(9)-O(1)	-109.7(2)
C(3)-C(4)-C(9)-O(1)	17.7(2)
O(3)-C(4)-C(9)-C(8)	-104.1(2)
C(5)-C(4)-C(9)-C(8)	9.3(2)
C(3)-C(4)-C(9)-C(8)	136.66(19)
C(9')-O(1')-C(2')-C(3')	11.6(2)
O(1')-C(2')-C(3')-C(4')	14.2(2)

C(2')-C(3')-C(4')-O(4')	82.5(2)
C(2')-C(3')-C(4')-C(5')	-156.74(19)
C(2')-C(3')-C(4')-C(9')	-32.5(2)
C(4)-O(3)-C(5')-C(4')	-126.44(18)
C(4)-O(3)-C(5')-C(6')	0.9(2)
O(4')-C(4')-C(5')-O(3)	-42.4(2)
C(3')-C(4')-C(5')-O(3)	-161.04(18)
C(9')-C(4')-C(5')-O(3)	81.9(2)
O(4')-C(4')-C(5')-C(6')	-161.91(18)
C(3')-C(4')-C(5')-C(6')	79.5(2)
C(9')-C(4')-C(5')-C(6')	-37.6(3)
O(3)-C(5')-C(6')-C(5)	28.0(2)
C(4')-C(5')-C(6')-C(5)	148.55(18)
O(3)-C(5')-C(6')-C(7')	-88.4(2)
C(4')-C(5')-C(6')-C(7')	32.1(3)
C(4)-C(5)-C(6')-C(5')	-44.12(19)
C(6)-C(5)-C(6')-C(5')	-163.57(18)
C(4)-C(5)-C(6')-C(7')	77.5(2)
C(6)-C(5)-C(6')-C(7')	-41.9(2)
C(5')-C(6')-C(7')-O(7')	-154.07(17)
C(5)-C(6')-C(7')-O(7')	93.94(19)
C(5')-C(6')-C(7')-C(8')	-36.3(2)
C(5)-C(6')-C(7')-C(8')	-148.27(18)
C(5')-C(6')-C(7')-C(8)	91.2(2)
C(5)-C(6')-C(7')-C(8)	-20.8(2)
C(7)-C(8)-C(7')-O(7')	-42.6(2)
C(9)-C(8)-C(7')-O(7')	-157.05(17)
C(7)-C(8)-C(7')-C(8')	-161.41(18)
C(9)-C(8)-C(7')-C(8')	84.1(2)
C(7)-C(8)-C(7')-C(6')	71.2(2)
C(9)-C(8)-C(7')-C(6')	-43.2(2)
O(7')-C(7')-C(8')-C(9')	165.16(18)
C(6')-C(7')-C(8')-C(9')	48.8(2)
C(8)-C(7')-C(8')-C(9')	-76.7(2)
C(2')-O(1')-C(9')-C(8')	86.7(2)
C(2')-O(1')-C(9')-C(4')	-32.8(2)
C(7')-C(8')-C(9')-O(1')	-169.25(17)
C(7')-C(8')-C(9')-C(4')	-54.3(2)

O(4')-C(4')-C(9')-O(1')	-70.8(2)
C(5')-C(4')-C(9')-O(1')	165.40(17)
C(3')-C(4')-C(9')-O(1')	40.6(2)
O(4')-C(4')-C(9')-C(8')	170.83(17)
C(5')-C(4')-C(9')-C(8')	47.1(2)
C(3')-C(4')-C(9')-C(8')	-77.8(2)

Symmetry transformations used to generate equivalent atoms:

Table S4. Crystal data and structure refinement for (−)-incarvilleatone [(−)-**1**].

Identification code	(−)-incarvilleatone [(−)-**1**]	
Empirical formula	$C_{16} H_{20} O_6$	
Formula weight	308.32	
Temperature	100(2) K	
Wavelength	0.71073 Å	
Crystal system	Orthorhombic	
Space group	$P2_12_12_1$	
Unit cell dimensions	a = 6.16240(10) Å	α = 90°.
	b = 9.1961(2) Å	β = 90°.
	c = 24.1420(6) Å	γ = 90°.
Volume	1368.13(5) $Å^3$	
Z	4	
Density (calculated)	1.497 Mg/m^3	
Absorption coefficient	0.114 mm^{-1}	
F(000)	656	
Crystal size	0.180 x 0.150 x 0.050 mm^3	
Theta range for data collection	3.364 to 30.512°.	
Index ranges	-8<=h<=7, -13<=k<=13, -34<=l<=34	
Reflections collected	21030	
Independent reflections	4099 [R(int) = 0.0257]	
Completeness to theta = 25.242°	96.9 %	
Absorption correction	Semi-empirical from equivalents	
Max. and min. transmission	0.994 and 0.980	
Refinement method	Full-matrix least-squares on F^2	
Data / restraints / parameters	4099 / 0 / 201	
Goodness-of-fit on F^2	1.059	
Final R indices [I>2sigma(I)]	R1 = 0.0274, wR2 = 0.0724	

R indices (all data)	R1 = 0.0279, wR2 = 0.0728
Absolute structure parameter	0.01(14)
Extinction coefficient	n/a
Largest diff. peak and hole	0.317 and -0.156 e.Å^{-3}

Table S5. Bond lengths [Å] and angles [°] for (−)-incarvilleatone [(−)-**1**].

O(1)-C(9)	1.4238(14)
O(1)-C(2)	1.4458(15)
C(2)-C(3)	1.529(2)
C(2)-H(2A)	0.9900
C(2)-H(2B)	0.9900
C(3)-C(4)	1.5312(16)
C(3)-H(3A)	0.9900
C(3)-H(3B)	0.9900
C(4)-O(3)	1.4336(14)
C(4)-C(5)	1.5285(17)
C(4)-C(9)	1.5502(17)
O(3)-C(5')	1.4437(15)
C(5)-C(6)	1.5280(17)
C(5)-C(6')	1.5444(16)
C(5)-H(5)	1.0000
C(6)-C(7)	1.5042(19)
C(6)-H(6A)	0.9900
C(6)-H(6B)	0.9900
C(7)-O(2)	1.2199(16)
C(7)-C(8)	1.5182(17)
C(8)-C(9)	1.5246(15)
C(8)-C(7')	1.5513(16)
C(8)-H(8)	1.0000
C(9)-H(9)	1.0000
O(1')-C(9')	1.4431(14)
O(1')-C(2')	1.4459(16)
C(2')-C(3')	1.5243(18)
C(2')-H(2'A)	0.9900
C(2')-H(2'B)	0.9900
C(3')-C(4')	1.5279(17)
C(3')-H(3'A)	0.9900

C(3')-H(3'B)	0.9900
C(4')-O(4')	1.4269(14)
C(4')-C(5')	1.5220(16)
C(4')-C(9')	1.5305(17)
O(4')-H(4'A)	0.8400
C(5')-C(6')	1.5409(16)
C(5')-H(5')	1.0000
C(6')-C(7')	1.5569(16)
C(6')-H(6')	1.0000
C(7')-O(7')	1.4323(14)
C(7')-C(8')	1.5351(16)
O(7')-H(7'A)	0.8400
C(8')-C(9')	1.5297(16)
C(8')-H(8'A)	0.9900
C(8')-H(8'B)	0.9900
C(9')-H(8')	1.0000
C(9)-O(1)-C(2)	103.93(9)
O(1)-C(2)-C(3)	103.99(10)
O(1)-C(2)-H(2A)	111.0
C(3)-C(2)-H(2A)	111.0
O(1)-C(2)-H(2B)	111.0
C(3)-C(2)-H(2B)	111.0
H(2A)-C(2)-H(2B)	109.0
C(2)-C(3)-C(4)	103.23(10)
C(2)-C(3)-H(3A)	111.1
C(4)-C(3)-H(3A)	111.1
C(2)-C(3)-H(3B)	111.1
C(4)-C(3)-H(3B)	111.1
H(3A)-C(3)-H(3B)	109.1
O(3)-C(4)-C(5)	103.84(9)
O(3)-C(4)-C(3)	111.43(10)
C(5)-C(4)-C(3)	118.78(10)
O(3)-C(4)-C(9)	109.71(9)
C(5)-C(4)-C(9)	109.27(9)
C(3)-C(4)-C(9)	103.73(10)
C(4)-O(3)-C(5')	108.17(9)
C(6)-C(5)-C(4)	112.86(11)

C(6)-C(5)-C(6')	114.06(10)
C(4)-C(5)-C(6')	97.93(9)
C(6)-C(5)-H(5)	110.5
C(4)-C(5)-H(5)	110.5
C(6')-C(5)-H(5)	110.5
C(7)-C(6)-C(5)	110.05(10)
C(7)-C(6)-H(6A)	109.7
C(5)-C(6)-H(6A)	109.7
C(7)-C(6)-H(6B)	109.7
C(5)-C(6)-H(6B)	109.7
H(6A)-C(6)-H(6B)	108.2
O(2)-C(7)-C(6)	123.04(12)
O(2)-C(7)-C(8)	124.02(12)
C(6)-C(7)-C(8)	112.93(10)
C(7)-C(8)-C(9)	106.71(9)
C(7)-C(8)-C(7')	104.73(10)
C(9)-C(8)-C(7')	111.07(9)
C(7)-C(8)-H(8)	111.3
C(9)-C(8)-H(8)	111.3
C(7')-C(8)-H(8)	111.3
O(1)-C(9)-C(8)	110.25(9)
O(1)-C(9)-C(4)	105.66(9)
C(8)-C(9)-C(4)	111.26(10)
O(1)-C(9)-H(9)	109.9
C(8)-C(9)-H(9)	109.9
C(4)-C(9)-H(9)	109.9
C(9')-O(1')-C(2')	109.00(9)
O(1')-C(2')-C(3')	106.92(10)
O(1')-C(2')-H(2'A)	110.3
C(3')-C(2')-H(2'A)	110.3
O(1')-C(2')-H(2'B)	110.3
C(3')-C(2')-H(2'B)	110.3
H(2'A)-C(2')-H(2'B)	108.6
C(2')-C(3')-C(4')	101.03(10)
C(2')-C(3')-H(3'A)	111.6
C(4')-C(3')-H(3'A)	111.6
C(2')-C(3')-H(3'B)	111.6
C(4')-C(3')-H(3'B)	111.6

H(3'A)-C(3')-H(3'B)	109.4
O(4')-C(4')-C(5')	109.64(9)
O(4')-C(4')-C(3')	105.53(9)
C(5')-C(4')-C(3')	114.46(10)
O(4')-C(4')-C(9')	110.50(10)
C(5')-C(4')-C(9')	114.74(10)
C(3')-C(4')-C(9')	101.38(9)
C(4')-O(4')-H(4'A)	109.5
O(3)-C(5')-C(4')	107.91(9)
O(3)-C(5')-C(6')	105.95(9)
C(4')-C(5')-C(6')	118.65(10)
O(3)-C(5')-H(5')	108.0
C(4')-C(5')-H(5')	108.0
C(6')-C(5')-H(5')	108.0
C(5')-C(6')-C(5)	99.67(9)
C(5')-C(6')-C(7')	115.40(9)
C(5)-C(6')-C(7')	109.11(9)
C(5')-C(6')-H(6')	110.7
C(5)-C(6')-H(6')	110.7
C(7')-C(6')-H(6')	110.7
O(7')-C(7')-C(8')	107.40(9)
O(7')-C(7')-C(8)	102.05(9)
C(8')-C(7')-C(8)	114.99(10)
O(7')-C(7')-C(6')	109.78(9)
C(8')-C(7')-C(6')	112.60(9)
C(8)-C(7')-C(6')	109.41(9)
C(7')-O(7')-H(7'A)	109.5
C(9')-C(8')-C(7')	117.99(10)
C(9')-C(8')-H(8'A)	107.8
C(7')-C(8')-H(8'A)	107.8
C(9')-C(8')-H(8'B)	107.8
C(7')-C(8')-H(8'B)	107.8
H(8'A)-C(8')-H(8'B)	107.1
O(1')-C(9')-C(8')	108.45(9)
O(1')-C(9')-C(4')	104.71(9)
C(8')-C(9')-C(4')	112.46(10)
O(1')-C(9')-H(8')	110.4
C(8')-C(9')-H(8')	110.4

C(4')-C(9')-H(8')	110.4

Symmetry transformations used to generate equivalent atoms:

Table S6. Torsion angles [°] for (−)-incarvilleatone [(−)-**1**].

C(9)-O(1)-C(2)-C(3)	-45.10(12)
O(1)-C(2)-C(3)-C(4)	32.29(13)
C(2)-C(3)-C(4)-O(3)	109.35(11)
C(2)-C(3)-C(4)-C(5)	-130.06(11)
C(2)-C(3)-C(4)-C(9)	-8.61(12)
C(5)-C(4)-O(3)-C(5')	28.29(11)
C(3)-C(4)-O(3)-C(5')	157.29(10)
C(9)-C(4)-O(3)-C(5')	-88.41(11)
O(3)-C(4)-C(5)-C(6)	-166.67(9)
C(3)-C(4)-C(5)-C(6)	68.95(14)
C(9)-C(4)-C(5)-C(6)	-49.66(13)
O(3)-C(4)-C(5)-C(6')	-46.32(11)
C(3)-C(4)-C(5)-C(6')	-170.69(11)
C(9)-C(4)-C(5)-C(6')	70.70(10)
C(4)-C(5)-C(6)-C(7)	55.64(13)
C(6')-C(5)-C(6)-C(7)	-54.98(13)
C(5)-C(6)-C(7)-O(2)	179.43(12)
C(5)-C(6)-C(7)-C(8)	0.46(13)
O(2)-C(7)-C(8)-C(9)	122.71(13)
C(6)-C(7)-C(8)-C(9)	-58.33(13)
O(2)-C(7)-C(8)-C(7')	-119.45(13)
C(6)-C(7)-C(8)-C(7')	59.51(11)
C(2)-O(1)-C(9)-C(8)	159.62(10)
C(2)-O(1)-C(9)-C(4)	39.30(12)
C(7)-C(8)-C(9)-O(1)	-53.45(13)
C(7')-C(8)-C(9)-O(1)	-167.03(9)
C(7)-C(8)-C(9)-C(4)	63.44(12)
C(7')-C(8)-C(9)-C(4)	-50.14(12)
O(3)-C(4)-C(9)-O(1)	-137.26(9)
C(5)-C(4)-C(9)-O(1)	109.50(10)
C(3)-C(4)-C(9)-O(1)	-18.12(12)
O(3)-C(4)-C(9)-C(8)	103.08(11)

C(5)-C(4)-C(9)-C(8)	-10.16(12)
C(3)-C(4)-C(9)-C(8)	-137.78(10)
C(9')-O(1')-C(2')-C(3')	6.24(13)
O(1')-C(2')-C(3')-C(4')	-29.19(12)
C(2')-C(3')-C(4')-O(4')	-75.64(11)
C(2')-C(3')-C(4')-C(5')	163.72(10)
C(2')-C(3')-C(4')-C(9')	39.63(11)
C(4)-O(3)-C(5')-C(4')	130.10(10)
C(4)-O(3)-C(5')-C(6')	2.05(12)
O(4')-C(4')-C(5')-O(3)	43.76(12)
C(3')-C(4')-C(5')-O(3)	162.10(10)
C(9')-C(4')-C(5')-O(3)	-81.26(12)
O(4')-C(4')-C(5')-C(6')	164.14(10)
C(3')-C(4')-C(5')-C(6')	-77.52(13)
C(9')-C(4')-C(5')-C(6')	39.12(15)
O(3)-C(5')-C(6')-C(5)	-30.75(11)
C(4')-C(5')-C(6')-C(5)	-152.13(11)
O(3)-C(5')-C(6')-C(7')	85.89(11)
C(4')-C(5')-C(6')-C(7')	-35.48(15)
C(6)-C(5)-C(6')-C(5')	164.92(10)
C(4)-C(5)-C(6')-C(5')	45.46(10)
C(6)-C(5)-C(6')-C(7')	43.62(13)
C(4)-C(5)-C(6')-C(7')	-75.83(11)
C(7)-C(8)-C(7')-O(7')	46.50(11)
C(9)-C(8)-C(7')-O(7')	161.32(9)
C(7)-C(8)-C(7')-C(8')	162.40(9)
C(9)-C(8)-C(7')-C(8')	-82.78(12)
C(7)-C(8)-C(7')-C(6')	-69.74(11)
C(9)-C(8)-C(7')-C(6')	45.09(12)
C(5')-C(6')-C(7')-O(7')	155.98(10)
C(5)-C(6')-C(7')-O(7')	-92.84(11)
C(5')-C(6')-C(7')-C(8')	36.39(13)
C(5)-C(6')-C(7')-C(8')	147.56(9)
C(5')-C(6')-C(7')-C(8)	-92.80(11)
C(5)-C(6')-C(7')-C(8)	18.38(12)
O(7')-C(7')-C(8')-C(9')	-165.69(10)
C(8)-C(7')-C(8')-C(9')	81.52(13)
C(6')-C(7')-C(8')-C(9')	-44.72(14)

C(2')-O(1')-C(9')-C(8')	-100.65(11)
C(2')-O(1')-C(9')-C(4')	19.61(13)
C(7')-C(8')-C(9')-O(1')	163.87(10)
C(7')-C(8')-C(9')-C(4')	48.57(14)
O(4')-C(4')-C(9')-O(1')	74.25(11)
C(5')-C(4')-C(9')-O(1')	-161.18(10)
C(3')-C(4')-C(9')-O(1')	-37.28(11)
O(4')-C(4')-C(9')-C(8')	-168.20(9)
C(5')-C(4')-C(9')-C(8')	-43.63(13)
C(3')-C(4')-C(9')-C(8')	80.27(11)

Symmetry transformations used to generate equivalent atoms:

Table S7. Crystal data and structure refinement for (+)-incarvilleatone [(+)-**1**]

Identification code	(+)-incarvilleatone [(+)-**1**]	
Empirical formula	C16 H20 O6	
Formula weight	308.32	
Temperature	100(2) K	
Wavelength	0.71073 Å	
Crystal system	Orthorhombic	
Space group	$P2_12_12_1$	
Unit cell dimensions	a = 6.1648(2) Å	α = 90°.
	b = 9.2025(2) Å	β = 90°.
	c = 24.1266(5) Å	γ = 90°.
Volume	1368.74(6) $Å^3$	
Z	4	
Density (calculated)	1.496 Mg/m^3	
Absorption coefficient	0.114 mm^{-1}	
F(000)	656	
Crystal size	0.170 x 0.090 x 0.040 mm^3	
Theta range for data collection	3.364 to 33.769°.	
Index ranges	-9<=h<=9, -12<=k<=14, -37<=l<=31	
Reflections collected	28955	
Independent reflections	5436 [R(int) = 0.0233]	
Completeness to theta = 25.242°	97.2 %	
Absorption correction	Semi-empirical from equivalents	

Max. and min. transmission	0.995 and 0.981
Refinement method	Full-matrix least-squares on F^2
Data / restraints / parameters	5436 / 0 / 201
Goodness-of-fit on F^2	1.090
Final R indices [I>2sigma(I)]	R1 = 0.0278, wR2 = 0.0739
R indices (all data)	R1 = 0.0285, wR2 = 0.0743
Absolute structure parameter	0.13(12)
Extinction coefficient	n/a
Largest diff. peak and hole	0.389 and -0.174 e.Å^{-3}

Table S8. Bond lengths [Å] and angles [°] for (+)-incarvilleatone [(+)-**1**].

O(1)-C(9)	1.4235(12)
O(1)-C(2)	1.4443(13)
C(2)-C(3)	1.5295(17)
C(2)-H(2A)	0.9900
C(2)-H(2B)	0.9900
O(2)-C(7)	1.2218(13)
C(3)-C(4)	1.5309(13)
C(3)-H(3A)	0.9900
C(3)-H(3B)	0.9900
C(4)-O(3)	1.4332(12)
C(4)-C(5)	1.5298(14)
C(4)-C(9)	1.5505(14)
O(3)-C(5')	1.4439(12)
C(5)-C(6)	1.5287(14)
C(5)-C(6')	1.5459(13)
C(5)-H(5)	1.0000
C(6)-C(7)	1.5040(15)
C(6)-H(6A)	0.9900
C(6)-H(6B)	0.9900
C(7)-C(8)	1.5181(14)
C(8)-C(9)	1.5248(13)
C(8)-C(7')	1.5528(14)
C(8)-H(8)	1.0000
C(9)-H(9)	1.0000
O(1')-C(9')	1.4428(12)
O(1')-C(2')	1.4461(14)

C(2')-C(3')	1.5233(15)
C(2')-H(2'A)	0.9900
C(2')-H(2'B)	0.9900
C(3')-C(4')	1.5273(14)
C(3')-H(3'A)	0.9900
C(3')-H(3'B)	0.9900
C(4')-O(4')	1.4274(12)
C(4')-C(5')	1.5225(14)
C(4')-C(9')	1.5311(14)
O(4')-H(4'A)	0.8400
C(5')-C(6')	1.5407(13)
C(5')-H(5')	1.0000
C(6')-C(7')	1.5577(13)
C(6')-H(6')	1.0000
C(7')-O(7')	1.4328(11)
C(7')-C(8')	1.5354(13)
O(7')-H(7'A)	0.8400
C(8')-C(9')	1.5303(13)
C(8')-H(8'A)	0.9900
C(8')-H(8'B)	0.9900
C(9')-H(9')	1.0000
C(9)-O(1)-C(2)	103.97(8)
O(1)-C(2)-C(3)	104.04(8)
O(1)-C(2)-H(2A)	110.9
C(3)-C(2)-H(2A)	110.9
O(1)-C(2)-H(2B)	110.9
C(3)-C(2)-H(2B)	110.9
H(2A)-C(2)-H(2B)	109.0
C(2)-C(3)-C(4)	103.19(8)
C(2)-C(3)-H(3A)	111.1
C(4)-C(3)-H(3A)	111.1
C(2)-C(3)-H(3B)	111.1
C(4)-C(3)-H(3B)	111.1
H(3A)-C(3)-H(3B)	109.1
O(3)-C(4)-C(5)	103.84(8)
O(3)-C(4)-C(3)	111.51(8)
C(5)-C(4)-C(3)	118.70(8)

O(3)-C(4)-C(9)	109.78(8)
C(5)-C(4)-C(9)	109.20(7)
C(3)-C(4)-C(9)	103.73(8)
C(4)-O(3)-C(5')	108.21(7)
C(6)-C(5)-C(4)	112.86(9)
C(6)-C(5)-C(6')	114.08(8)
C(4)-C(5)-C(6')	97.87(7)
C(6)-C(5)-H(5)	110.5
C(4)-C(5)-H(5)	110.5
C(6')-C(5)-H(5)	110.5
C(7)-C(6)-C(5)	110.08(8)
C(7)-C(6)-H(6A)	109.6
C(5)-C(6)-H(6A)	109.6
C(7)-C(6)-H(6B)	109.6
C(5)-C(6)-H(6B)	109.6
H(6A)-C(6)-H(6B)	108.2
O(2)-C(7)-C(6)	123.10(10)
O(2)-C(7)-C(8)	123.94(10)
C(6)-C(7)-C(8)	112.95(8)
C(7)-C(8)-C(9)	106.69(7)
C(7)-C(8)-C(7')	104.74(8)
C(9)-C(8)-C(7')	111.05(8)
C(7)-C(8)-H(8)	111.4
C(9)-C(8)-H(8)	111.4
C(7')-C(8)-H(8)	111.4
O(1)-C(9)-C(8)	110.27(8)
O(1)-C(9)-C(4)	105.70(7)
C(8)-C(9)-C(4)	111.34(8)
O(1)-C(9)-H(9)	109.8
C(8)-C(9)-H(9)	109.8
C(4)-C(9)-H(9)	109.8
C(9')-O(1')-C(2')	108.93(8)
O(1')-C(2')-C(3')	106.97(8)
O(1')-C(2')-H(2'A)	110.3
C(3')-C(2')-H(2'A)	110.3
O(1')-C(2')-H(2'B)	110.3
C(3')-C(2')-H(2'B)	110.3
H(2'A)-C(2')-H(2'B)	108.6

C(2')-C(3')-C(4')	101.09(8)
C(2')-C(3')-H(3'A)	111.6
C(4')-C(3')-H(3'A)	111.6
C(2')-C(3')-H(3'B)	111.6
C(4')-C(3')-H(3'B)	111.6
H(3'A)-C(3')-H(3'B)	109.4
O(4')-C(4')-C(5')	109.62(8)
O(4')-C(4')-C(3')	105.54(8)
C(5')-C(4')-C(3')	114.51(8)
O(4')-C(4')-C(9')	110.51(8)
C(5')-C(4')-C(9')	114.76(8)
C(3')-C(4')-C(9')	101.32(8)
C(4')-O(4')-H(4'A)	109.5
O(3)-C(5')-C(4')	107.92(8)
O(3)-C(5')-C(6')	105.96(7)
C(4')-C(5')-C(6')	118.65(8)
O(3)-C(5')-H(5')	108.0
C(4')-C(5')-H(5')	108.0
C(6')-C(5')-H(5')	108.0
C(5')-C(6')-C(5)	99.72(7)
C(5')-C(6')-C(7')	115.41(7)
C(5)-C(6')-C(7')	109.08(8)
C(5')-C(6')-H(6')	110.7
C(5)-C(6')-H(6')	110.7
C(7')-C(6')-H(6')	110.7
O(7')-C(7')-C(8')	107.44(7)
O(7')-C(7')-C(8)	102.00(7)
C(8')-C(7')-C(8)	114.97(8)
O(7')-C(7')-C(6')	109.79(7)
C(8')-C(7')-C(6')	112.62(7)
C(8)-C(7')-C(6')	109.40(7)
C(7')-O(7')-H(7'A)	109.5
C(9')-C(8')-C(7')	117.93(8)
C(9')-C(8')-H(8'A)	107.8
C(7')-C(8')-H(8'A)	107.8
C(9')-C(8')-H(8'B)	107.8
C(7')-C(8')-H(8'B)	107.8
H(8'A)-C(8')-H(8'B)	107.2

O(1')-C(9')-C(8')	108.48(8)
O(1')-C(9')-C(4')	104.75(8)
C(8')-C(9')-C(4')	112.47(8)
O(1')-C(9')-H(9')	110.3
C(8')-C(9')-H(9')	110.3
C(4')-C(9')-H(9')	110.3

Symmetry transformations used to generate equivalent atoms:

Table S9. Torsion angles [°] for (+)-incarvilleatone [(+)-**1**].

C(9)-O(1)-C(2)-C(3)	45.02(10)
O(1)-C(2)-C(3)-C(4)	-32.32(10)
C(2)-C(3)-C(4)-O(3)	-109.36(9)
C(2)-C(3)-C(4)-C(5)	130.04(9)
C(2)-C(3)-C(4)-C(9)	8.72(10)
C(5)-C(4)-O(3)-C(5')	-28.38(9)
C(3)-C(4)-O(3)-C(5')	-157.34(8)
C(9)-C(4)-O(3)-C(5')	88.28(9)
O(3)-C(4)-C(5)-C(6)	166.65(7)
C(3)-C(4)-C(5)-C(6)	-68.91(12)
C(9)-C(4)-C(5)-C(6)	49.59(10)
O(3)-C(4)-C(5)-C(6')	46.30(8)
C(3)-C(4)-C(5)-C(6')	170.74(9)
C(9)-C(4)-C(5)-C(6')	-70.76(8)
C(4)-C(5)-C(6)-C(7)	-55.61(10)
C(6')-C(5)-C(6)-C(7)	54.94(11)
C(5)-C(6)-C(7)-O(2)	-179.42(10)
C(5)-C(6)-C(7)-C(8)	-0.46(11)
O(2)-C(7)-C(8)-C(9)	-122.74(11)
C(6)-C(7)-C(8)-C(9)	58.31(10)
O(2)-C(7)-C(8)-C(7')	119.45(11)
C(6)-C(7)-C(8)-C(7')	-59.50(9)
C(2)-O(1)-C(9)-C(8)	-159.60(8)
C(2)-O(1)-C(9)-C(4)	-39.14(10)
C(7)-C(8)-C(9)-O(1)	53.55(10)
C(7')-C(8)-C(9)-O(1)	167.12(8)
C(7)-C(8)-C(9)-C(4)	-63.46(10)

C(7')-C(8)-C(9)-C(4)	50.11(10)
O(3)-C(4)-C(9)-O(1)	137.21(8)
C(5)-C(4)-C(9)-O(1)	-109.55(8)
C(3)-C(4)-C(9)-O(1)	17.93(10)
O(3)-C(4)-C(9)-C(8)	-103.04(9)
C(5)-C(4)-C(9)-C(8)	10.20(10)
C(3)-C(4)-C(9)-C(8)	137.68(8)
C(9')-O(1')-C(2')-C(3')	-6.11(11)
O(1')-C(2')-C(3')-C(4')	29.12(10)
C(2')-C(3')-C(4')-O(4')	75.67(9)
C(2')-C(3')-C(4')-C(5')	-163.68(8)
C(2')-C(3')-C(4')-C(9')	-39.58(9)
C(4)-O(3)-C(5')-C(4')	-130.00(8)
C(4)-O(3)-C(5')-C(6')	-1.93(10)
O(4')-C(4')-C(5')-O(3)	-43.67(10)
C(3')-C(4')-C(5')-O(3)	-162.04(8)
C(9')-C(4')-C(5')-O(3)	81.36(9)
O(4')-C(4')-C(5')-C(6')	-164.06(8)
C(3')-C(4')-C(5')-C(6')	77.57(11)
C(9')-C(4')-C(5')-C(6')	-39.03(12)
O(3)-C(5')-C(6')-C(5)	30.66(9)
C(4')-C(5')-C(6')-C(5)	152.06(9)
O(3)-C(5')-C(6')-C(7')	-85.99(9)
C(4')-C(5')-C(6')-C(7')	35.41(12)
C(6)-C(5)-C(6')-C(5')	-164.82(8)
C(4)-C(5)-C(6')-C(5')	-45.40(8)
C(6)-C(5)-C(6')-C(7')	-43.49(11)
C(4)-C(5)-C(6')-C(7')	75.93(8)
C(7)-C(8)-C(7')-O(7')	-46.44(9)
C(9)-C(8)-C(7')-O(7')	-161.24(7)
C(7)-C(8)-C(7')-C(8')	-162.35(8)
C(9)-C(8)-C(7')-C(8')	82.85(10)
C(7)-C(8)-C(7')-C(6')	69.78(9)
C(9)-C(8)-C(7')-C(6')	-45.01(10)
C(5')-C(6')-C(7')-O(7')	-156.07(8)
C(5)-C(6')-C(7')-O(7')	92.70(9)
C(5')-C(6')-C(7')-C(8')	-36.41(11)
C(5)-C(6')-C(7')-C(8')	-147.63(8)

C(5')-C(6')-C(7')-C(8)	92.76(9)
C(5)-C(6')-C(7')-C(8)	-18.46(10)
O(7')-C(7')-C(8')-C(9')	165.79(8)
C(8)-C(7')-C(8')-C(9')	-81.46(11)
C(6')-C(7')-C(8')-C(9')	44.76(12)
C(2')-O(1')-C(9')-C(8')	100.61(9)
C(2')-O(1')-C(9')-C(4')	-19.71(10)
C(7')-C(8')-C(9')-O(1')	-163.98(8)
C(7')-C(8')-C(9')-C(4')	-48.59(11)
O(4')-C(4')-C(9')-O(1')	-74.20(9)
C(5')-C(4')-C(9')-O(1')	161.24(8)
C(3')-C(4')-C(9')-O(1')	37.31(9)
O(4')-C(4')-C(9')-C(8')	168.18(8)
C(5')-C(4')-C(9')-C(8')	43.61(11)
C(3')-C(4')-C(9')-C(8')	-80.32(9)

Symmetry transformations used to generate equivalent atoms:

Crystal data of (±)-**4** $C_{16}H_{20}O_6$, M = 308.32, colorless block, 0.46 x 0.41 x 0.32 mm^3, monoclinic, space group $P2_1/n$, a = 10.1468(10) Å, b = 10.0947(11) Å, c = 14.8924(14) Å, β = 108.972(4)°, V = 1442.5(3) Å^3, Z = 4, T = 150(2) K, $2\theta_{max}$=50.00°, D_{calc} (g cm^{-3}) = 1.420, $F(000)$ = 656, μ (mm^{-1}) = 0.109, 5958 reflections collected, 1356 unique reflections (R_{int}=0.0249), 1187 observed ($I > 2\sigma(I)$) reflections, multi-scan absorption correction, T_{min} = 0.952, T_{max} = 0.966, 210 refined parameters, 12 restraints, S = 1.091, $R1$ = 0.0358, $wR2$ = 0.0813 (all data R = 0.0436, $wR2$ = 0.0863), maximum and minimum residual electron densities; $\Delta\rho_{max}$ = 0.167, $\Delta\rho_{min}$= -0.179 (eÅ^{-3}).
